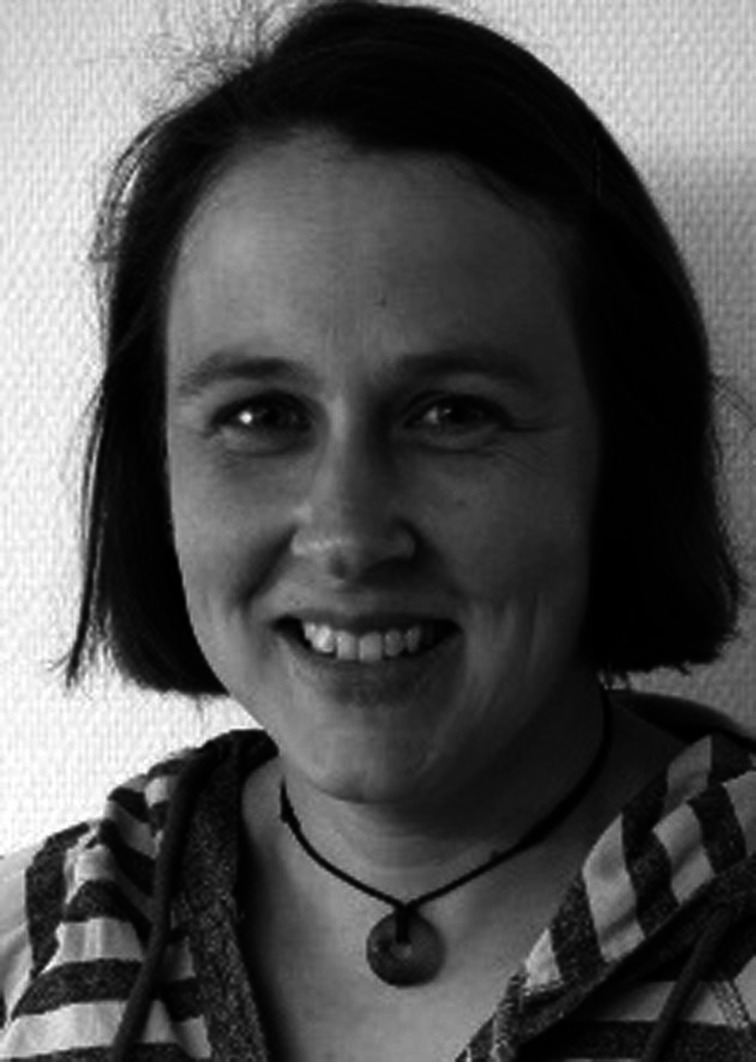# Methyltransferases: Functions and Applications

**DOI:** 10.1002/cbic.202200212

**Published:** 2022-07-05

**Authors:** Eman Abdelraheem, Benjamin Thair, Romina Fernández Varela, Emely Jockmann, Désirée Popadić, Helen C. Hailes, John M. Ward, Adolfo M. Iribarren, Elizabeth S. Lewkowicz, Jennifer N. Andexer, Peter‐Leon Hagedoorn, Ulf Hanefeld

**Affiliations:** ^1^ Biocatalysis Department of Biotechnology Delft University of Technology Van der Maasweg 9 2629 HZ Delft (The Netherlands; ^2^ Department of Chemistry University College London 20 Gordon Street London WC1H 0AJ UK; ^3^ Laboratorio de Biotransformaciones y Química de Ácidos Nucleicos Universidad Nacional de Quilmes Roque S. Peña 352 B1876BXD Bernal Argentina; ^4^ Institute of Pharmaceutical Sciences University of Freiburg Albertstr. 25 79104 Freiburg Germany; ^5^ Department of Biochemical Engineering Bernard Katz Building University College London London WC1E 6BT UK

**Keywords:** biocatalysis, enzymes, methyltransferases, *S*-adenosyl-l-methionine

## Abstract

In this review the current state‐of‐the‐art of *S*‐adenosylmethionine (SAM)‐dependent methyltransferases and SAM are evaluated. Their structural classification and diversity is introduced and key mechanistic aspects presented which are then detailed further. Then, catalytic SAM as a target for drugs, and approaches to utilise SAM as a cofactor in synthesis are introduced with different supply and regeneration approaches evaluated. The use of SAM analogues are also described. Finally *O*‐, *N*‐, *C*‐ and *S*‐MTs, their synthetic applications and potential for compound diversification is given.

## Graphical abstract

**Methyltransferases** are enzymes that will in the future enable clean and green alkylation of amino and hydroxy groups as well as reactive carbon atoms. These enzymes, their cofactor SAM (*S*‐adenosylmethionine) and the opportunities’ they offer for new S_N_2 chemistries are highlighted.

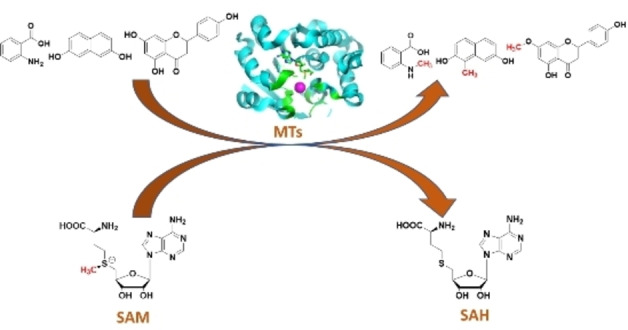

## 1 Introduction

The methylation of hydroxyl‐ and amino‐groups, thiols and reactive carbons is an essential synthetic reaction, yet at the same time traditional synthetic methods use very toxic reagents. These reactions, and indeed all nucleophilic substitution reactions, have been identified as particularly problematic on an industrial scale. In 2007, leading pharmaceutical companies highlighted this type of reaction as a priority for replacement with catalytic and environmentally benign approaches.^[1]^

Methylation is a universal reaction in all living organisms. This transformation, catalysed to a large extent by *S*‐adenosylmethionine (SAM, AdoMet)‐dependent methyltransferases (MTs), plays an important role in different biological processes^[2]^ such as cell signalling,^[3]^ as membrane components^[4]^ and pigments,^[5]^ and for the expression, structure, and function of biological molecules such as proteins and DNA/RNA.^[6]^ In general, methylation is an important step for the diversification of the structure of natural products in biosynthetic pathways, which can be utilised in chemo‐, regio‐ and stereospecific synthesis. These are the features required for selective synthesis, and therefore, MTs are potentially industrially relevant for the synthesis of active pharmaceutical ingredients (APIs).[^1^, ^7^]

**Scheme 1:**
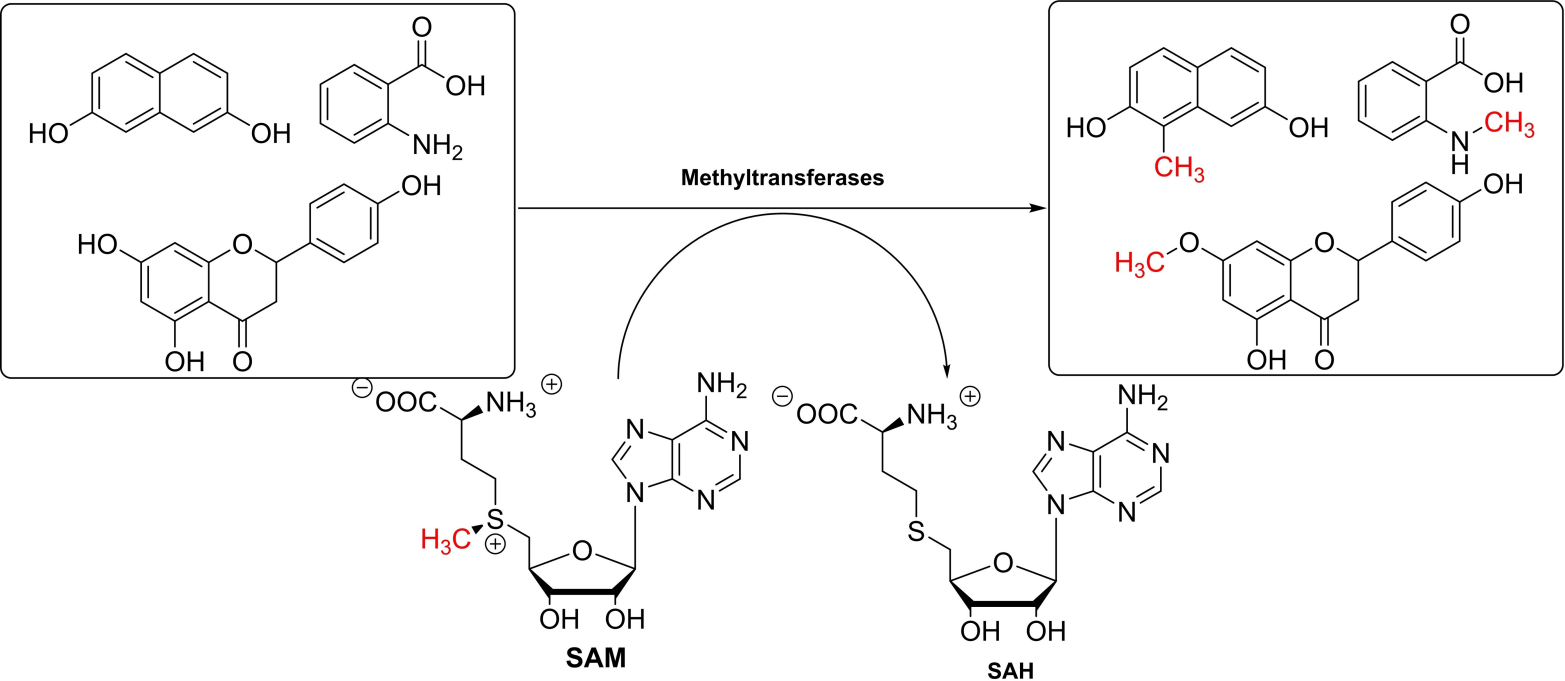
SAM‐dependent methyltransferases (MTs) have many potential applications in synthesis (SAH: *S*‐adenosylhomocysteine).

In biological systems, MTs^[8]^ use the co‐substrate SAM as an electrophilic methyl donor (Scheme 1). Several problems limiting the use of SAM as a cofactor in synthetic methylation applications need to be overcome. In this review, we will highlight the different classes of MTs (see section 2.), catalytic mechanisms of the methylation (see section 3.), SAM cofactor supply and regeneration, methyl acceptor diversity (see section 4.), and different applications of the methylation reaction for the production of APIs (see section 5.). The review focusses on MTs following an S_N_2 or S_N_2‐like mechanism, as shown in Scheme 1, as these are currently the main group studied for application in chemical synthesis. MTs from the radical SAM family are another large and multifaceted group of methyl‐transferring enzymes, which have been reviewed in detail elsewhere and are only mentioned briefly.^[9]^

## 2 Methyltransferase Classification

Enzymes using SAM as a methyl donor have been present since the last universal common ancestor. Those primordial MTs have expanded and diversified over millions of years, while convergence has pulled together unrelated families from the several independent re‐discoveries of this reaction.^[10]^ The present evolutionary picture is therefore complex and, to date, incomplete. Nonetheless, the bioinformatic work of Schubert *et al*. showed that the structures of SAM‐dependent MTs coalesce into fivefold‐classes.^[11]^ These classes share little in terms of amino acid sequence, mode of SAM binding, tertiary structure and quaternary behaviour. However, the recurrence of structural motifs within the groups has illuminated their key features. Furthermore, the variety of forms achieving the same catalytic goal points to a plasticity in SAM‐dependent MTs, perhaps allowed by the energetic favourability of the methylation itself.^[11]^

**Figure 1:**
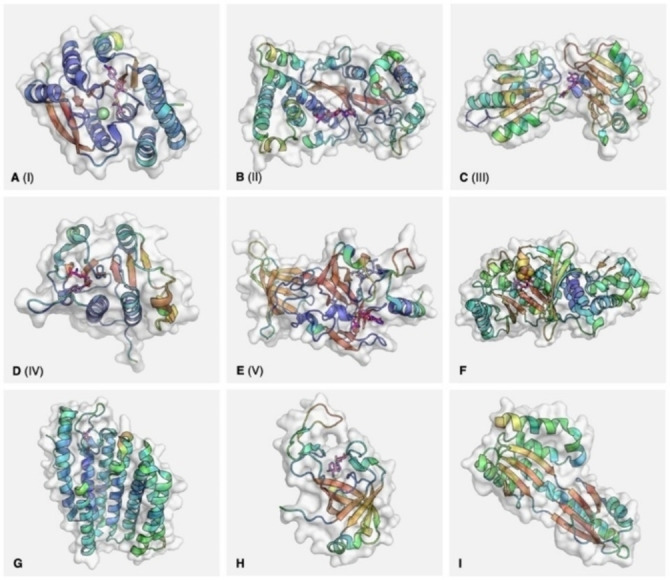
Representative structures for the known classes of methyltransferase. PDB codes: A – 6LFE (catechol‐*O*‐methyltransferase); B – 1MSK (methionine synthase, reactivation domain); C – 1CBF (cobalt precorrin‐4‐methyltransferase); D – 1MXI (tRNA (cytidine(34)‐2’‐*O*)‐methyltransferase); E – 1O9S (SET7/9); F – 3RFA (RlmN); G – 5VG9 (isoprenylcysteine carboxyl methyltransferase); H – 2NV4 (AF0241); I – 1TLJ (Taw3). Secondary structures represented within the molecular surface as cartoons, coloured by B factor: blue (high) to red (low) for α‐helices, the reverse for β‐sheets. Cofactor represented as magenta sticks. For E (1O9S), histone peptide represented as blue sticks. For F (3RFA), iron‐sulfur cluster represented as spheres.

The first MT structure to be solved was of HhaI MT (DNA cytosine‐5‐MT).^[12]^ Its topology defines class I, which has remained the largest group, and is otherwise referred to as the Rossmann‐like structure. Class I MTs feature an alternating α/β sequence, which folds into a seven‐strand sheet sandwiched by helices on each side (Figure 1A).^[12]^ SAM binds in an elongated conformation at the C‐terminal ends of β1 and β2. Many characteristic features of the class are located in this region. They include a loosely conserved GxGxG motif between β1 and αA to accommodate the adenine moiety, a hydrogen bonding residue between β2 and αB to coordinate the ribose moiety, and an acidic residue within β1, which may make water‐assisted hydrogen bonds with the methionine.[^10a^, ^11^] However, the neighbouring substrate‐binding and catalytic regions vary significantly. This reflects the diverse functions of the class, which comprise methylations of small molecules,^[13]^ cofactors,^[14]^ antibiotics^[15]^ and biomacromolecules.[^12^, ^16^] Substrate selectivity is sometimes enforced by additional domains, which may be appended to the Rossmann fold,[^13a^, ^13c^] or embedded within it.[^12^, ^13b^]

In contrast to class I, class II MTs have as yet only one known function: to reactivate oxidised cobalamin.^[17]^ They form one domain of the modular enzyme methionine synthase, which uses cobalamin as a cofactor. In the normal reaction cycle, the cofactor alternates between methylcobalamin and cob(I)alamin states. If the cobalt ion is oxidised, however, the inactive cob(II)alamin is reversibly reduced by flavodoxin and presented to the MT domain by a conformational change. The cob(I)alamin thus generated is ‘captured’ by attack of its lone pair on the reactive methyl of SAM, regenerating methylcobalamin and restarting the reaction cycle.^[18]^ The central feature is a long antiparallel β‐sheet with a sharp kink near one end (Figure 1B). Helices surround the sheet above and below and extend beyond both ends, forming a horseshoe‐like topology. As with class I, SAM binds in an elongated conformation, in a groove by the inner edge of the central β‐sheet.^[18]^

Class III MTs are dimeric, with the active site situated between the two domains (Figure 1C).^[19]^ The lobes are similar in composition, both featuring central β‐sheets flanked by α‐helices. However, their topologies are distinct (3‐2‐4‐1‐5 for the N‐terminal sheet, 1‐2‐5‐3‐4 for the C‐terminal), and the C‐terminal domain has a β‐turn between strands 4 and 5 to give a mostly antiparallel sheet.^[19]^ In solution, the proteins dimerise across the bridge between the two domains, such that the sheets are continuous across the monomers. SAM binds in a deep pocket at the bridge, near the N‐terminal end of αE. Unlike in previous classes, it takes on a tightly folded conformation which exposes the activated methyl group to the exterior.[^11^, ^19^] The main targets of class III MTs are tetrapyrroles; the prototypical example, cobalt‐precorrin‐4 MT, is involved in anaerobic vitamin B12 biosynthesis.^[20]^ The enzyme accommodates its substrate in a groove along the N‐terminal domain. The site runs across the C‐terminal ends of the parallel β‐sheet, and is walled by loops emanating from those strands.^[19]^ Some mechanistic understanding of class III MTs has been derived from another example: SAM urophorphyrinogen III methyltransferase (SUMT; see also section 3.).^[21]^ In that enzyme the first reaction between the tetrapyrrole and SAM is assisted by a residue acting as a general base, as in many other MTs. A subsequent rearrangement, thought to be aided by another basic residue, then primes the methylation at a second position.^[21]^ Other class III examples with similar structures include sirohaem synthase,^[22]^ which operates in the same biosynthetic pathway as SUMT, and diphthine^[22]^ synthase which modifies a histidine residue on elongation factor 2.^[16a]^

The class IV MTs are encompassed by the SPOUT enzyme superfamily, named after the bacterial tRNA MTs SpoU (now TrmH) and TrmD.^[23]^ They have roles in post‐transcriptional modification of t‐ and r‐RNAs, and are the second‐most populous group after class I.^[23]^ The class IV structure is analogous to class I, with a 6‐strand, parallel β‐sheet flanked by α‐helices, although five of those helices sit on one side of the sheet and two on the other (Figure 1D). The crystal structure of TrmL shows SAH in a bent conformation, in a pocket formed by the ends of β3, β4 and β5 and their associated loops.^[24]^ Part of the cofactor binding site is a rare ‘knot’ topology, formed by the loop between β6 and αG passing through the loop between β4 and αH. This knot is the strongest characteristic feature of class IV.^[25]^ Regions outside the motif, however, have low conservation even between members,^[23]^ and may facilitate interactions with substrates or other proteins.^[25]^ The catalytic mechanism of SPOUTs is in some cases unclear, but is thought to be assisted by a basic residue for both *N*‐ and *O*‐methylation.^[26]^ Dimerisation was originally thought necessary for the catalysis. However, recent work has also demonstrated the activity of class IV monomers.^[27]^

The final class are the SET domains (suppressor of variegation, enhancer of zeste and trithorax).^[28]^ They function to methylate histone lysines and other proteins important for transcriptional regulation across all forms of life, as well as RuBisCo.^[29]^ The superfamily seems to have evolved through domain duplication in eukaryotes, before laterally transferring to bacteria.^[10a]^ The typical structure is relatively complex, comprising four α‐helices embedded in a sequence of twisted β‐sheets (Figure 1E). A pseudo‐knot is formed by the loop prior to the C‐terminal helix, though with a different topology to that in class IV.[^28a^, ^30^] In a crystal structure of SET7/9, SAM binds near this knot, in a channel at the surface of the protein. The histone binds on the opposite face, with the target lysine side chain reaching through a narrow channel between the faces to approach the SAM methyl from behind.^[28a]^ The cofactor is thought to be held in its tightly folded conformation by hydrogen bonding of the methionine amine to a conserved asparagine, with the methyl transfer catalysed by a tyrosine residue.^[30]^ As with other classes, the SET domain itself is often flanked by poorly‐conserved but essential pre‐ and post‐SET regions that direct its specificity.^[11]^

In the years since Schubert *et al*. classification, a number of SAM‐dependent MT families with structures outside these groups have been characterised:

- Radical SAM MTs, feature a catalytic domain with an alpha/beta TIM barrel structure (Figure 1F).^[31]^ Many enzymes use the 5’‐dA⋅ intermediate generated from SAM, but only a small number of these methylate their product. The mechanism of methylation varies and has been described elsewhere.[^9b^, ^9c^, ^11^, ^32^, ^33^, ^36^, ^37^]
- Transmembrane MTs include isoprenylcysteine carboxyl methyltransferase (ICMT) as a well‐characterised example. ICMT performs the final step in prenylcysteine modification of proteins. In eukaryotes, its 6 to 8 α‐helices span the endoplasmic reticulum membrane, with the sheltered binding site for SAM protruding into the cytoplasm (Figure 1G).^[34]^ The substrate binds in a nearby cleft which begins in the cytoplasm and dives across the protein surface into the membrane.^[34a]^ Arginine residues in the active site have been implicated in positioning the substrate carboxy for nucleophilic attack on the SAM methyl group, while others appear to stabilise the transition state.^[34]^ A prokaryotic analogue MaICMT (or MaMTase) has a similar structure, with 5 transmembrane helices and a cytosolic cofactor binding site, but currently has no identified substrates.^[35]^
- β‐Barrel MTs are exemplified by the tRNA methyltransferase TrmO.^[36]^ The crystal structure of a homologue from *Archaeoglobus fulgidus* shows a tight β‐barrel formed of six antiparallel strands (Figure 1H).^[37]^ At the N‐terminal end of the barrel, according to the direction of the β1, an extended helix crosses the circular face to connect β2 and β3. At the C‐terminal end, three loops together form a large, lopsided V‐shape, with SAM binding in a tight pocket within the V‐shaped tip. The enzyme forms a dimer in solution, and the key residues in each monomer's cofactor pocket include Met57 and Leu133 to sandwich the adenine, Gln22 and Arg82 to hydrogen bond the methionine ammonium and Lys122, reaching across from the other subunit's β6, to hydrogen bond the carboxyl group.^[37]^ Though the MT activity of the enzyme towards tRNA substrates has been confirmed, the binding site of the substrate has not, so the mechanism has not yet been elucidated.^[36]^
- Sso0622‐like MTs include the *S. cerevisiae* protein TWY3 and its *Sulfolobus solfataricus* homologue Taw3.^[38]^ A crystal structure of Taw3 shows two 4‐strand, antiparallel β‐sheets, connected end‐to‐end by disordered loops (Figure 1I). Both sheets lie against and parallel to the long spine of αE and are flanked by further helices.^[38a]^ The protein is actually an amalgamation and extension of two recognised folds. The sheet formed by β2, β3, β4 and β8, as well as αC beside it and part of that spine, constitute a RAGNYA domain typically involved in interactions with other macromolecules.^[39]^ Strands β5, β6 and β7 and nearby αD form an SHS2 domain, which can take on a variety of functions.^[40]^ The authors of the study were unable to demonstrate activity of the purified protein *in vitro*, nor able to obtain a structure co‐crystallised with the cofactor. However, computational docking of the cofactor implicated a groove at the surface of the N‐terminal extension, formed by αA, αB and β1, as the binding site.^[38a]^

Given the number of MT genes with as yet unknown structures, it is possible there are further structural classes of SAM‐dependent MTs waiting to be found, either concentrated within clades or spread across all domains of life.^[41]^

## 3 Catalytic Mechanisms of SAM‐Dependent MTs

The SAM‐dependent methylation reaction of *O*‐, *N*‐, *C*‐ or *S*‐ occurs via a nucleophilic substitution S_N_2 mechanism,^[42]^ where the methyl acceptor in the substrate and methyl donor atom in SAM are presented in a linear position (around 3 Å to the methyl carbon and 4 Å between the acceptor and sulfonium group) as required by the methyl transfer reaction.^[43]^ Generally, the reaction starts with the reorientation of adjacent residues or flexible loops of the enzyme upon binding of the substrates. This is followed by the second step, the nucleophilic attack on the activated methyl group of SAM resulting in cleavage of the C−S bond. For the S_N_2 and S_N_2‐like methylation reaction catalysed by MTs three catalytic mechanisms have been described. In addition a radical‐based SAM mechanism exists[^9a^, ^9b^, ^44^] as indicated above (see section 2): a recent review covers this topic and it will therefore not be discussed here.[^9b^, ^33b^, ^33c^, ^33d^, ^33e^]

- The proximity and desolvation mechanism was reported in the SABATH (Salicylic Acid, Benzoic Acid, THeobromine synthase) family of plant MTs.^[3]^ In the catalytic reaction, the active site residues of the enzyme do not directly participate in the mechanism but the enzyme pocket helps to bring the acceptor and donor in close proximity with optimal orientation for the nucleophilic substitution. Solvating water is eliminated from the interface of the donor and the acceptor (desolvation), increasing the reactivity of the nucleophile and electrophile.^[3]^ For carminomycin‐4‐*O*‐MT (DnrK) this was demonstrated in the biosynthesis of daunorubicin. In the active site of DnrK, the methylated oxygen of the product is proximal to the sulfonium group of SAM (around 4.13 Å). Tyr142 is positioned such that it might activate O‐4; however, mutagenesis of Tyr142 did not have a substantial effect on catalysis of the reaction (Figure 2
  **Figure 2:**
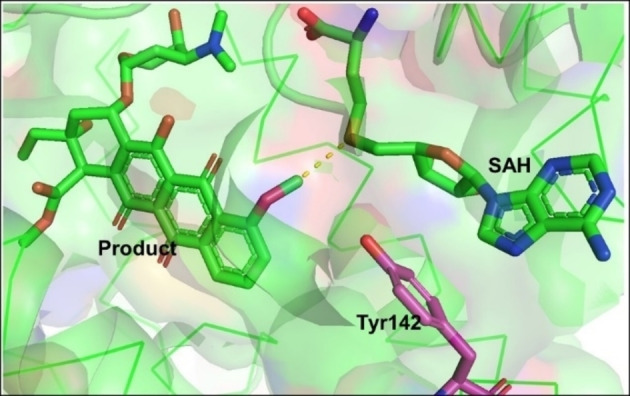
The proximity and desolvation mechanism of DnrK (PDB ID: 1TW2) enables the selective methylation in the daunorubicin biosynthesis.
  )^[45]^ supporting the proximity and desolvation mechanism.
- Other MTs utilise an acid/base‐mediated trans‐methylation mechanism depending on catalytic residues such as arginine or histidine. These residues act as a base to deprotonate the substrate for nucleophilic attack on the reactive methyl group of SAM. This is for instance essential in the biosynthesis of precorrin‐2 by the action of SUMTs as indicated in section 2, in this specific case NirE. The amino acid residues Arg111 and Glu114 are involved in the catalytic mechanism of *C*‐methylation of uroporphyrinogen III.^[46]^ Arg111 was found to be an essential base to deprotonate C‐20 in the substrate, this was followed by methylation at C‐2, while residue Glu114 is important for the correct orientation of Arg111. Also, important hydrogen‐bonds of SAH and uroporphyrinogen III with Met186 are shown in Figure 3
  **Figure 3:**
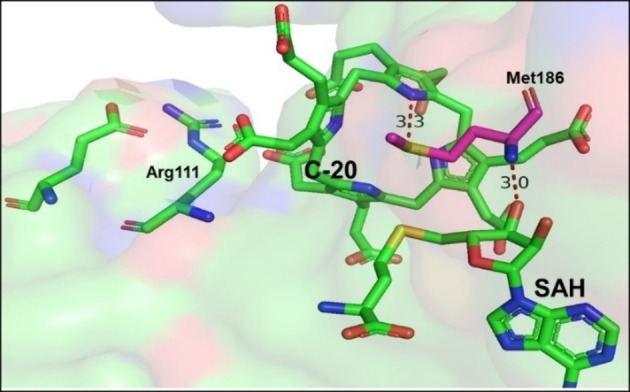
Active site of NirE with Arg111 as base for the protonation of the substrate. Hydrogen bonds between the SAH and the protein due to Met186 help to keep the two reacting molecules in place (PDB ID: 2YBQ).
  .
- Metal‐dependent mechanisms, which are almost exclusively found in phenolic *O*‐MTs in plants^[47]^ have been identified. Here a metal activates the phenol groups. For caffeoyl coenzyme A 3‐*O* MTs (CCoAOMT),^[48]^ Ca^2+^ in the active site activates the phenolic group by altering its pK_a_. The substrate can then form an oxyanion adjacent to the electron‐deficient methyl group on SAM, enhancing the subsequent selective methylation reaction (Figure 4
  **Figure 4:**
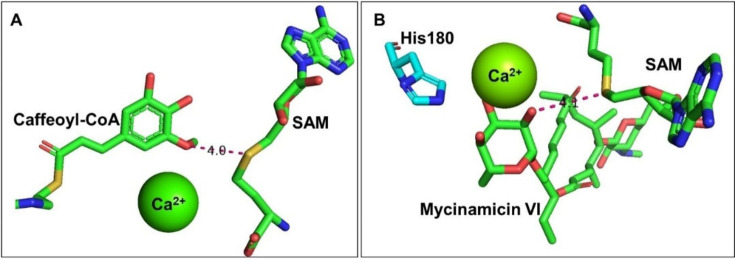
Metal‐dependent methylation catalysis mechanism for A) CCoAOMT (PDB ID: 1SUI) and B) Mycinamicin VI in the active site of MycE (PDB ID: 3SSN).
  A). Similarly, the methylation of mycinamicins^[49]^ employs Mg^2+^ as a divalent cation for substrate coordination and stabilisation of the hydroxylate intermediate, while a histidine residue acts as a general base for deprotonation of the methyl acceptor (Figure 4B). Here the remarkable selectivity of the MycE MT should be noted that discriminates between three sugar hydroxyl groups.

**Figure 5:**
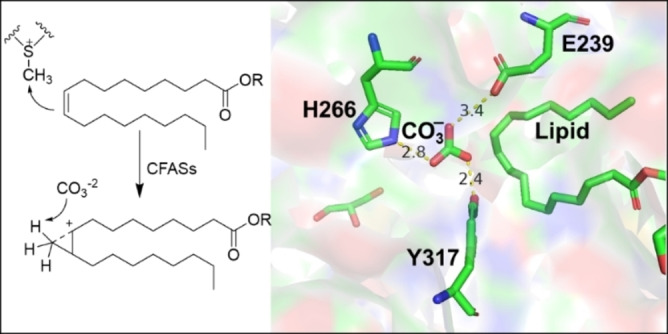
CFASs catalyses the formation of cyclopropane where carbonate ion is coordinated with labelled residues. The carbonate also acts as base (PDB ID: 6BQC).

Intriguingly, the enzymatic mechanism of SAM‐dependent methylation reactions can be more complicated when the substrates are alkenes. The formation of cyclopropane from lipophilic double bonds using cyclopropane fatty acid synthases (CFASs)^[50]^ is one of the examples which highlights this. Here, the alkene acts as a nucleophile to attack the methyl group in the SAM cofactor, forming a carbocation intermediate. Then, a carbonate ion in the active site of CFASs acts as base to deprotonate the methyl moiety of the carbocation intermediate to form the cyclopropane ring (Figure 5).

## 4 SAM

Variations of the cofactor structure have been described for many cofactors, including SAM. In addition to naturally occurring variations,^[51]^ cofactor analogues have been designed for a range of purposes.^[52]^ These include the generation of non‐functional derivatives acting as competitive inhibitors, or, when they bind covalently to the active site, irreversible inhibitors. Furthermore, cofactors have been redesigned towards less complex structures, with higher stability, or other modified properties. Last, but not least, in the case of group‐transferring cofactors such as SAM, analogues can be used to transfer non‐physiological residues onto the acceptor substrate.

### 4.1 SAM as target structure for APIs

**Figure 6:**
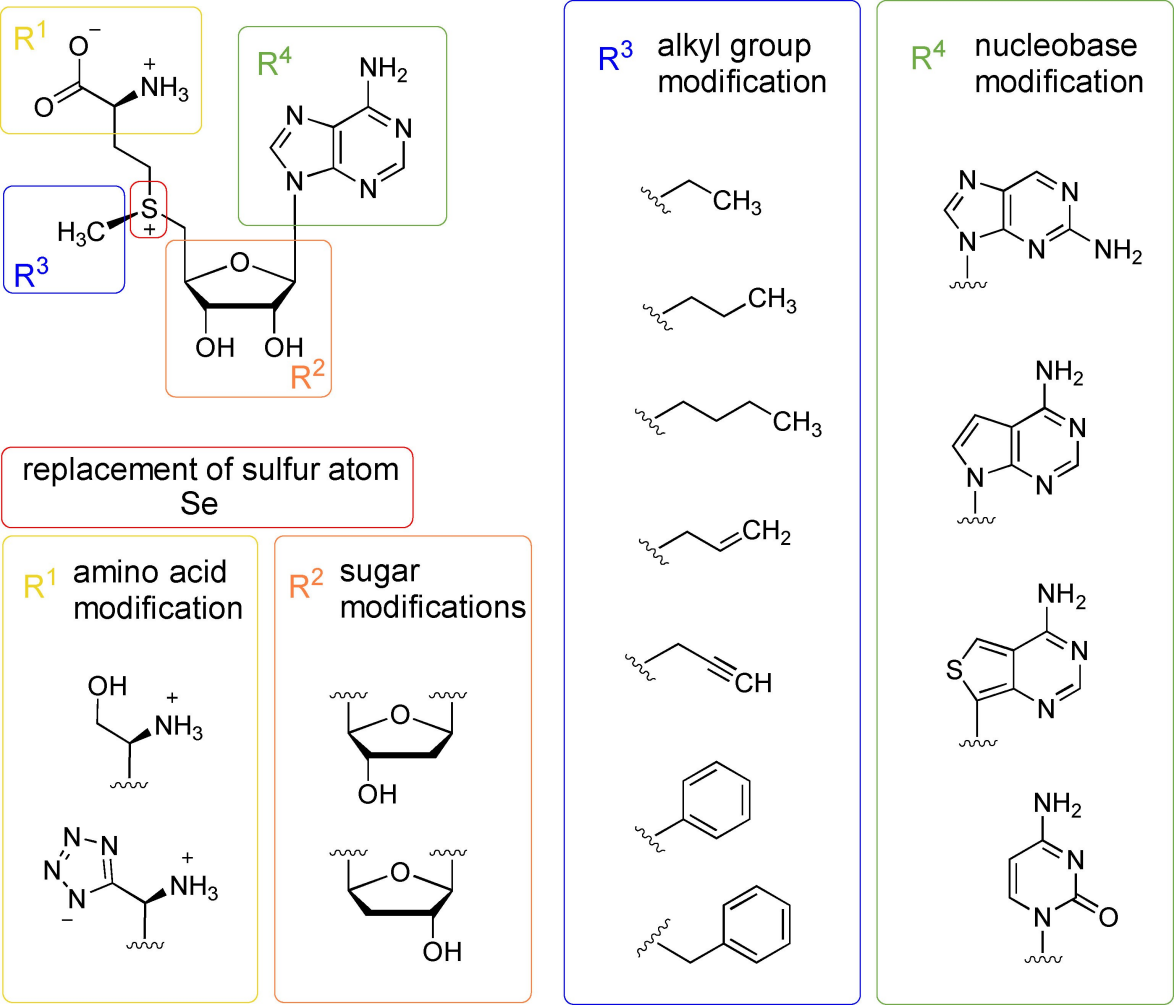
SAM modification on the alkyl group (blue), amino acid (yellow), sugar (orange), nucleobase (green) and replacement of the sulfur atom (red).

In general, SAM can be modified on all three parts of its structure: the amino acid chain, the sugar‐ and the nucleobase‐moiety (Figure 6). The alkyl residue of the sulfur atom (the group to be transferred), will be discussed separately below. SAM analogues, as well as SAH analogues, have been studied as small molecule inhibitors for MTs. As a result, SAM analogues are potential APIs for the pharmaceutical industry, e. g. for Parkinson's disease.[^53^, ^54^] The important role that methyl groups play in the regulation of the activity, selectivity, solubility, metabolism and pharmacokinetic/pharmacodynamic properties of biologically active molecules is well known and has been recently documented.^[55]^ In nature, methylation is used in all branches of metabolism and is often key to metabolic homeostasis by modulating various biological processes such as cell signalling and the biosynthesis of specialised metabolites.^[43]^ Methyl groups are introduced on a variety of substrates including histones, DNA, and RNA, a prominent example being protein arginine MT 5 (PRMT5) that is involved in genome organisation, regulation of transcription, and cell cycle, as well as spliceosome assembly.^[56]^

### 4.2 SAM‐dependent MTs in humans health and disease

Protein methylation is a fundamental epigenetic modification for the correct development of a wide range of biological processes. In humans, its dysregulation can lead to disorders such as cancer, neurodegenerative and cardiovascular diseases. In addition to SAM analogues used in Parkinson therapy, protein lysine and arginine MT inhibitors demonstrated therapeutic activities in human cancers.^[57]^ With the discovery of α‐*N*‐terminal MT 1 (NTMT1) and its physiological substrates, its possible participation in the response to DNA damage and cancer development was suggested.^[58]^ The protein methylation of an α‐*N* terminus by NTMT differs from the methylation of the side chain of lysine or arginine residues by protein lysine and arginine MT in that both, hydrophobicity and charge state, are altered under physiological conditions. NTMT1 was found to be overproduced in various tissues such as malignant melanoma and colorectal and brain cancer. Furthermore, the suppression of NTMT1 activity resulted in hypersensitivity to irradiation treatment of the breast cancer cell lines MCL‐7 and LCC9.^[59]^ Over four decades ago, *N*‐terminally methylated proteins such as myosin light chain LC‐1 and cytochrome c‐557 were reported to play a main role in the large macromolecular complexes, even though the methylated proteins have different overall functions.^[60]^ α‐*N*‐terminal methylation in protein‐DNA interactions has also been detected in interactions of the regulator of chromatin condensation 1 (RCC1) and of centromere protein A/B (CENP‐A/B) with chromatin.^[61]^ Furthermore, NTMTs are involved in protein stability where the main function of *N*‐α‐acetylation of cellular proteins is to prevent their degradation by the ubiquitin system.^[62]^ For the design of NTMT1 inhibitors, a bisubstrate strategy mimicking the ternary complex that occurs during catalysis has been applied. These inhibitors contain three components: a SAM analogue, a hexapeptide derived from the *N*‐terminal sequence of the NTMT1 substrate, and a linker.^[63]^

Given the difficulty of this type of bisubstrate inhibitors to penetrate cell membranes, Mackie *et al*. designed a new type of inhibitors taking advantage of the fact that the NTMT family has a unique peptide substrate binding site, favouring selectivity against other MTs.^[64]^ In this way, they synthesized the first potent and selective peptidomimetic inhibitor BM30 that presented a selectivity of more than 100 times to NTMT 1/2 among a panel of 41 MTs. More recently, a selective and potent NTMT1 inhibitor DC541 (IC_50_=0.34±0.02 μM) was obtained by introducing a naphthyl group at the *N*‐terminal region and an ortho‐aminobenzoic amide at the *C*‐terminal region of BM30. This compound inhibited the growth of human colorectal cancer cells without significant cytotoxicity up to 1 mM.^[65]^

One of the main functions of NTMTs is the methylation of alanine and glycine, where the trimethylation of alanine in human cells has an important effect on DNA damage repair. Damaged DNA binding protein 2 (DDB2) undergoes *N*‐methylation by NTMT in an Ala‐Pro‐Lys sequence. The methylated DDB2 then increases nuclear localisation, activates the ataxia telangiectasia mutation (ATM) and enhances the efficiency in cyclobutene pyrimidine dimer repair.^[66]^ Meanwhile, trimethylation of Gly has a positive effect on the functions of CENP‐A where human centromere has an important role in chromosome segregation to help genome stability. CENP‐A with an *N*‐terminal Gly‐Pro‐Arg sequence is subjected to α‐N‐methylation in the prenucleosomal form in cells. α‐Amino trimethylation of the CENP‐A *N* terminus is necessary for cell survival, increasing the CENPT and CENP‐I CCAN components at the centromere and helping in the formation of the bipolar spindle. The lack of this methylation could cause defects in chromosome segregation and cell death in the presence of p53.[^61b^, ^67^]

**Scheme 2:**
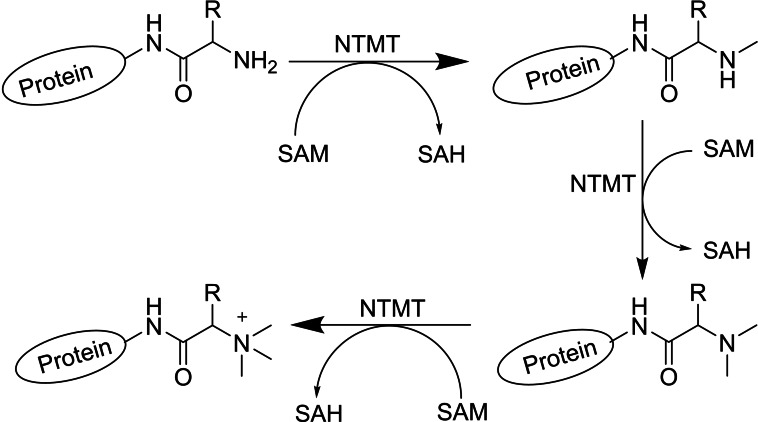
General strategy for protein methylation by NTMT. Protein *N*‐terminal methyltransferases (NTMTs) catalyse the transfer of methyl group from *S*‐adenosylmethionine (SAM) to the α‐amino group at the protein *N*‐terminus.

In related work, Chen and co‐workers reported that the *N*‐terminal methylation of serine or proline residues of RCC1 is a unique methylation process that participates in the interaction of RCC1 with chromosomes, which is critical for mitotic spindle assembly and function.^[68]^ In addition, α‐*N*‐methylation increased the binding of RCC1 to chromatin through electrostatic interactions with DNA.^[69]^ α‐*N*‐terminal MT 1 (NTMT1/NRMT1/METTL11A) catalyses the *N*‐terminal methylation of RCC1, explaining a new mechanism for the Ran GTPase activity which plays indispensable roles in nucleocytoplasmic transport and mitosis.^[70]^ Therefore, the discovery of α‐*N*‐terminal MT 1/2 (NTMT1 and NTMT2) and their physiological substrates has led to an explanation of the general role of α‐*N*‐terminal methylation in mediating DNA‐binding ability of the modified proteins. The NTMT1 and homologue NTMT2 can recognize any X‐P‐K/R consensus sequence in eukaryotic cells (X=S/P/A/G). Also, NTMT1 is able to methylate proteins starting with the amino acids S/P/A/G‐P‐K/R *in vivo* (Scheme 2).^[71]^ This means that there are more than 300 possible substrates for α‐*N*‐terminal MT 1 as reported based on the data bank analysis for NTMT1/2’s consensus sequences encoded on the human genome.^[72]^ Beside α‐*N*‐terminal methylation on the classical X‐P‐K/R sequence in eukaryotic cells, eukaryotic elongation factor 1 A (eEF1A) is a highly methylated protein in both yeast and humans which can facilitate translational elongation by delivering aminoacyl‐tRNAs to ribosomes. *Saccharomyces cerevisiae* YLR285W or elongation factor MT 7 (Efm7) methylates eEF1 A at both *N*‐terminal Gly1 and Lys2 residues. Nevertheless, this enzyme is unlike the other known eukaryotic *N*‐terminal MTs (NTMT1/2) as its substrate does not have an *N*‐terminal [A/P/S]‐P‐K motif at the *N*‐ termini.^[73]^

### 4.3 SAM and MTs in synthesis

For a successful wider application of SAM‐dependent MTs in chemical synthesis, two parameters are key: (i) to use MTs not only for the transfer of a methyl group but for alkyl groups in general, thereby expanding the scope of the enzymes (Figure 6). (ii) a robust regeneration or supply system for SAM; this should ideally be completely environmentally benign, otherwise issues with current traditional synthetic methylation methodology would merely be displaced.

#### 4.3.1 SAM analogues

In the context of synthetic applications, different aspects of SAM will be the focus here: SAM itself has a half‐life of 16 h at pH 8 and 37 °C due to non‐enzymatic degradation.^[74]^ This leads to an irreversible loss of the methyl group donor. An interesting application in biocatalysis are structural SAM analogues with prolonged half‐life under physiological conditions. They additionally could unlock a broad chemical space for the functionalisation of diverse substrates if the methyl group is exchanged with alternative groups (Figure 6).

Chemical synthesis of SAM analogues was first reported in the 1970s by alkylating SAH with alkyl halides, resulting in over 20 different SAM analogues including *Se*‐alkylated compounds.^[75]^ Synthesising these analogues enzymatically is easier, more sustainable and stereoselective. This stereoselectivity of the sulfur alkylation is important since MTs only accept the diastereomer with (*S*)‐configuration at the sulfur. The diastereomer with an (*R*)‐sulfur centre is a potential inhibitor for the reaction.^[76]^ In nature, SAM is synthesised by methionine adenosyltransferases (MATs) using methionine and ATP.^[77]^ *In vitro*, the reverse reaction of adenosyl‐chloride synthase (SalL) using methionine and 5’‐chloro‐‘5’‐deoxyadenosine (ClDA) as starting materials can also be exploited.^[78]^ The substrates of the two SAM‐forming enzymes can be chemically modified to enzymatically produce SAM analogues *in vitro*.^[79]^ The chemoenzymatic approach has the advantage that the synthesised SAM analogues can directly be utilised together with MTs for the alkylation. Here, a few examples will be highlighted, a more extensive overview is given in specialised reviews.[^52a^, ^53^, ^79a^, ^80^]

Different MATs from bacterial, eukaryotic, as well as archaeal origin have been used for *in situ* SAM synthesis; eukaryotic (e. g. human MAT II catalytic alpha subunit; hMAT2a) and archaeal variants (e. g. MATs from *Methanocaldococcus jannaschii* and *Sulfolobus solfataricus*) often show a broader substrate range than bacterial variants (e. g. MAT from *E. coli*).^[81]^ Engineering promising candidates by mutating amino acids in the active site lead to the acceptance of larger *S‐* or *Se*‐alkylated methionine analogues.^[82]^ Furthermore, some MATs have shown desirable properties for biocatalytic applications regarding temperature and organic solvent tolerance.^[83]^ Modified cofactors starting from methionine analogues, as well as ATP analogues, have been enzymatically synthesised. Recent examples include modifications of the terminal carboxylate/amine of methionine for future non‐native bio‐orthogonal applications^[82a]^ and of the nucleoside moiety for differential MTs targeting.^[84]^

The wild type SalL enzyme has a broad substrate range accepting methionine analogues with ethyl, propyl, butyl, allyl and benzyl groups with decreasing activity correlating to their size, but interestingly it did not accept propargyl nor phenethyl groups or polar analogues.^[85]^ In coupling the *in vitro* synthesis of the SAM analogues directly to a MT reaction that modifies a small peptide sequence, allylated as well as benzylated peptides were observed.^[85]^ Additionally, the synthetic SAM analogue S^th^AM (adenosine is replaced by a thieno[3,4‐*d*]pyrimidine‐based adenosine surrogate) was synthesised by SalL and used for DNA methylation *in vitro*.^[86]^ Recently, a tandem system for *C*‐methylation and *C*‐ethylation of coumarins was established.^[87]^

#### 4.3.2 Cofactor supply and regeneration

**Figure 7:**
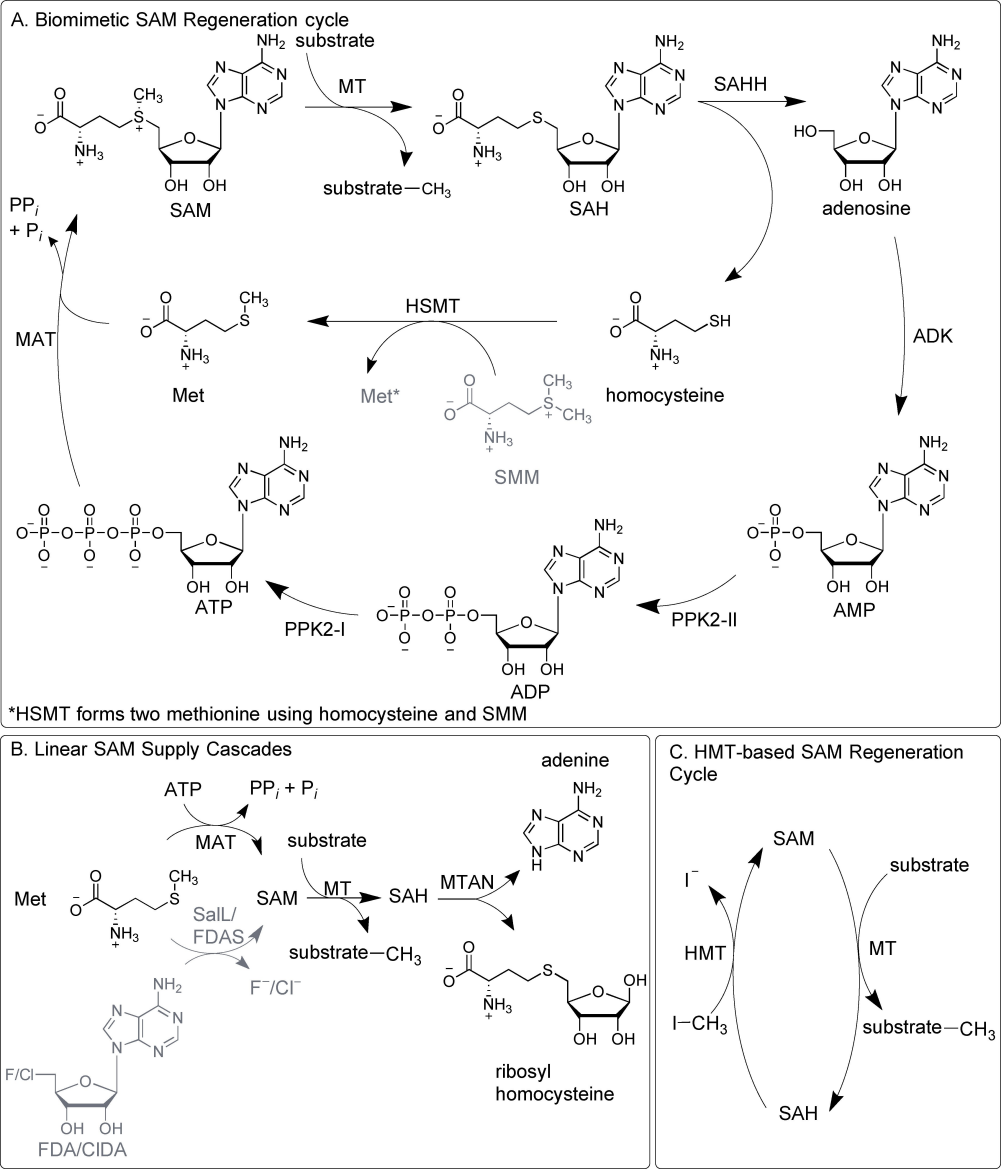
Enzymatic systems for SAM supply and regeneration. A. Cyclic regeneration system using seven enzymes starting from catalytic amounts of AMP and methionine. B. Linear enzyme cascades starting either from ATP and methionine or from ClDA/FDA and methionine. C. Two enzyme regeneration system starting from SAH. Enzymes: MAT=l‐methionine adenosyltransferase; MT=methyltransferase; MTAN=5’‐methylthioadenosine/*S*‐adenosyl‐l‐homocysteine nucleosidase; SAHH=*S*‐adenosyl‐l‐homocysteine hydrolase; ADK= adenosine kinase; PPK= polyphosphate kinase; HSMT=l‐homocysteine *S*‐methyltransferase: HMT=halide methyltransferase SalL=chlorinase FDAS=fluorinase. Substrates: ATP/ADP/AMP=adenosine 5’‐tri/di/monophosphate; Met=l‐methionine; SMM=*S*‐methyl‐l‐methionine; SAM=*S*‐adenosyl‐l‐methionine; SAH=*S*‐adenosyl‐l‐homocysteine. ClDA=5’‐chloro‐5’‐deoxyadenosine. FDA= 5’‐deoxy‐5’‐fluoroadenosine.

In addition to the *in situ* production of SAM, the degradation of SAH is a well‐established strategy. SAH inhibits SAM dependent MTs to various extents. It therefore needs to be removed to enable an efficient turnover of the methylation reaction.^[88]^ Two different enzymes can be used for degrading SAH: i) MTA/SAH nucleosidase (MTAN) that catalyses the irreversible cleavage to adenine and *S*‐ribosylhomocysteine (see Figure 7B),^[89]^ and ii) SAH hydrolase (SAHH) that hydrolyses SAH to adenosine and homocysteine in a reversible reaction using NAD^+^/NADH as a self‐regenerating cofactor (see Figure 7A). The equilibrium lies on the SAH side and the enzyme is therefore not suitable for a linear system.^[90]^

The enzymatic *in vitro* synthesis of SAM and its analogues is the first step of a supply or regeneration system for the cofactor. The chlorinase SalL naturally catalyses the formation of 5’‐chloro‐5’‐deoxyadenosine (ClDA), while fluorinases (FDAS) form 5’‐deoxy‐5’‐fluoroadenosine. In one of the first linear methylation cascades, those enzymes could be used to catalyse the reversed reaction to synthesise SAM *in vitro*.[^78^, ^91^] Alternatively, a three enzyme linear cascade utilising MTs in combination with MAT and MTAN starting from methionine and ATP has been used for the regioselective *O*‐methylation of catechol derivatives,^[92]^ and the production of *O*‐alkylated rebeccamycin derivatives.^[81d]^ These and similar systems are supply systems for SAM, where ATP is added in stoichiometric amounts (Figure 7B). To truly recycle SAM, ATP should be regenerated from the SAH degradation product. For this, adenosine kinase (ADK) is utilised for the first ATP‐dependent phosphorylation step yielding AMP.^[52a]^ AMP and ADP phosphorylation can be catalysed by various enzymes, e. g. polyphosphate kinases (PPK).^[93]^ Combining the three enzyme cascade (MAT, MT, MTAN) with the three kinases leads to an extended linear cascade for *in situ* SAM supply with adenine as an end product and conversions between 75–99 % for the *O*‐methylation of catechols and *N*‐methylation and *N*‐ethylation of anthranilic acid.^[94]^

Exchanging MTAN (yielding adenine) against SAHH (yielding adenosine) leads to a cyclic system, revealing the importance of the choice or approach for the SAH removal. The SAHH generated adenosine can be phosphorylated by ADK to re‐introduce it into the system. Overall, this results in a true SAM regeneration system (Figure 7A). After optimisation, this system achieves between 81 to 99 % conversion for the methylation of e. g. catechols and anthranilic acid with total turnover numbers (TTNs) of up to 200.[^94^, ^95^] In this cyclic regeneration system, homocysteine accumulates during the reactions. The free thiol group of this by‐product can interfere with several enzymes. Adding a homocysteine *S*‐methyltransferase (HSMT) and *S*‐methyl‐l‐methionine to the cycle removes homocysteine and also forms two molecules of methionine for further SAM syntheses. By adding this enzyme, the methionine concentration could be decreased to a catalytic amount;^[96]^ nevertheless, this is only possible if methionine is used as alkyl donor.

As an alternative, a one‐step regeneration system for SAM starting from SAH has been published with up to 500 TTNs using various *C*‐, *N*‐ and *O*‐MTs combined with halide MTs (HMT) using methyl iodide as stoichiometric methyl donor (Figure 7C).^[97]^ In comparison to a synthetic reaction using methyl iodide, the clear advantage is the selectivity of the methylation added by the MT. The recent adaption of the system to the corresponding bromo‐derivatives shows that improvements can be made, also chloro‐derivatives were accepted, albeit to a very small extend.^[98]^ Coupling the HMT/MT system to the PLP‐dependent oxidation of l‐amino acids and the following reduction of the methylated α‐keto acid resulted in the asymmetric β‐methylation of different l‐ and d‐α‐amino acids; as well as fluoromethylation reactions.^[99]^ By engineering an HMT from *Arabidopsis thaliana*, the formation of a cofactor analogue, using ethyl iodide could be increased five times compared to the reaction catalysed by the wild type enzyme. Also, SAM analogues with propyl or allyl chains could be formed and used for alkylation reactions catalysed by OMTs on a preparative scale.^[100]^ In another study, an HMT was used for SAM analogue formation in the synthesis of selective *N*‐alkylated pyrazoles using an engineered nicotinamide NMT from *Homo sapiens*. Different variants of this enzyme form possible regioisomers in different ratios and could, in some cases, reach a regioselectivity of over 99 %.^[101]^ To prevent enzymatic degradation of SAH to adenine and *S*‐ribosyl‐l‐homocysteine, the use of an MTAN deficient strain of *E. coli* as expression host is usually employed.[^97^, ^100^]

## 5 Methyltransferases Functional Classification and Their Applications in Drug Discovery

SAM dependent MTs can also be categorised by the different substrates used in methyl transfer reactions. DNA MTs (DNMTs) have become an important molecular target in the treatment and prevention of diseases.^[102]^ DNA/RNA methylation is a post‐replicative modification which occurs mostly in prokaryotic and eukaryotic genomes and its important biological functions include regulation of gene expression, preservation of chromosomal integrity and X‐chromosome inactivation. Methylation is an epigenetic modification which is catalysed by DNMTs such as DNMT1, DNMT2, and DNMT3. They all use SAM as a methyl donor and are therefore, as described above (section 4.2), of great importance in biological and medical research.

Another interesting type of MTs are protein MTs (e. g. histone MTs, see also 4.2). They are essential for epigenetic regulation of gene expression through the methylation reaction of histones and non‐histone proteins.^[103]^ The mis‐regulation of histone modifications causes the pathogenesis of cancer and developmental defects. Histone MTs modify lysine and arginine on histone tails, as discussed in 4.2.

The third type are non‐SAM dependent MTs. In this kind of reactions, B_12_‐cofactors (AdoCbl) and methylcobalamin (MeCbl) catalyse many biological processes, which are essential in the metabolism of microorganisms, animals and humans. These processes use methyl tetrahydrofolate, methanol, and methanethiol as methyl donors[^41^, ^104^] and are not further discussed here.

In the following part of the review, we will focus on the most abundant functional type of MTs that is of special interest for organic synthesis, **t**he natural product MTs (NPMTs).

### 5.1 Natural product methyltransferases (NPMTs)

NPMTs catalyse the selective addition of methyl groups to generate diverse natural substrates. Methyl groups can be added to S, N, O, or C atoms and they are classified by which of these atoms they modify; *O*‐MTs (OMT) represent the largest class (54 %) of the EC subclass.^[38b]^ The remaining enzymes methylate N (23 %), C (18 %), S (3 %) and others (2 %) such as NPMTs that accommodate other acceptors (e. g. halides).^[105]^

#### 5.1.1*. O*‐Methyltransferases (OMTs)

**Figure 8:**
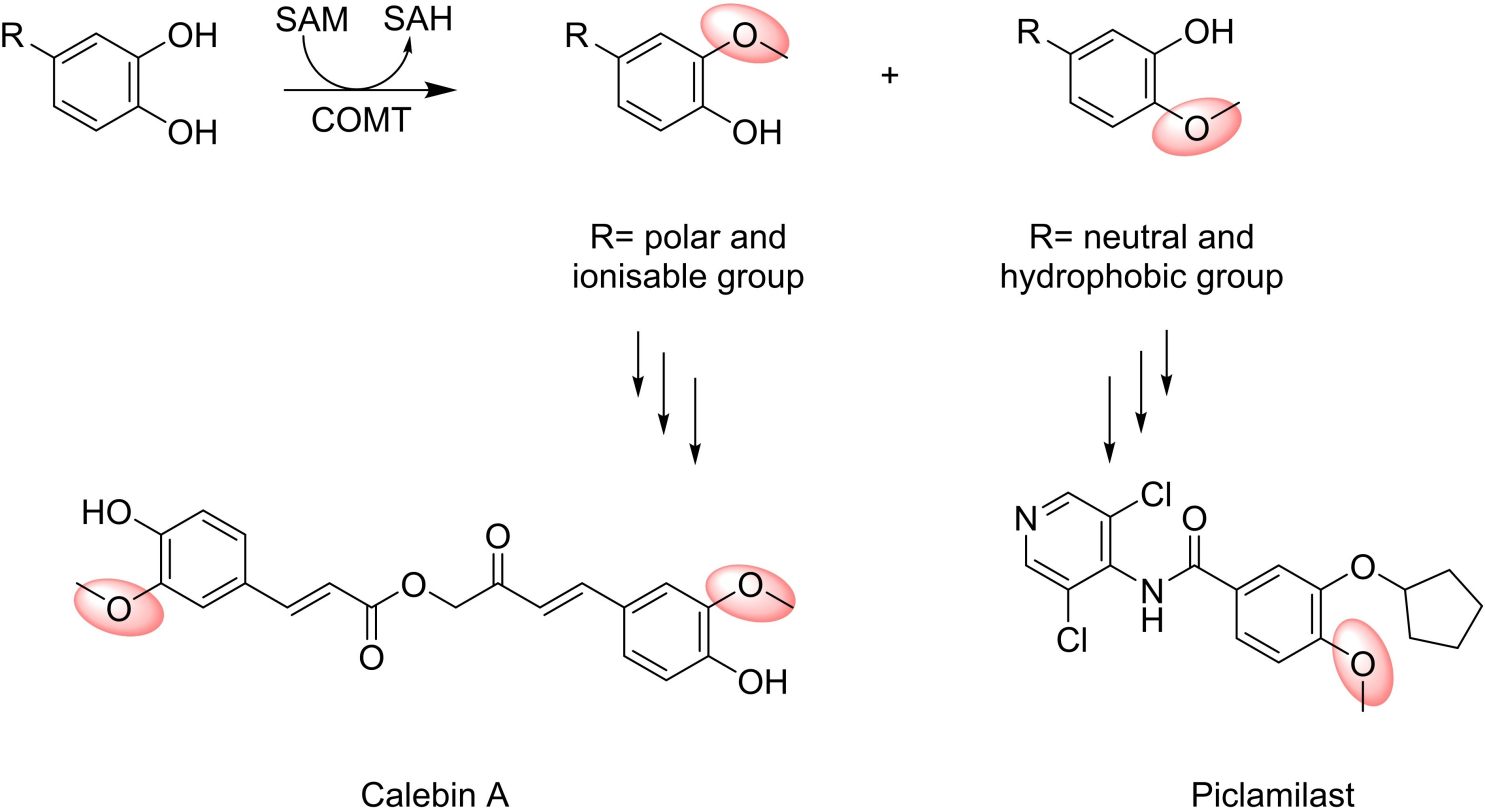
The regiospecific alkylation reaction for COMT.

Hydroxyl groups in phenolic compounds, such as in catechols, flavone derivatives, indole acetic acids, hydroxycinnamic acids, quercetagatein and CoA esters (Figure 8) are among the most abundant substrates for NPMTs. Their methylated products are used widely in the biotechnology and chemical industries.^[106]^ Some examples will be discussed in detail below.

Catechol‐*O‐*MT (COMT) is implicated in numerous neurological disorders such as Parkinson's disease. Consequently, COMT inhibition is a key strategy for the treatment of such diseases. In addition to catecholamines, other possible substrates of COMTs are hormones and xenobiotics that contain catechol structures.^[107]^ The polyphenols in coffee and tea and the oleosidic secoiridoids of the phenolic fraction of extra virgin olive oil have also been described as excellent substrates for COMT‐mediated *O‐*methylation and can behave as COMT inhibitors, preventing *O‐*methylation of a variety of catechol substrates.^[108]^ This dual mechanism can be interpreted as a direct competitive inhibition of the catalytic centre of COMTs, or as non‐competitive inhibition due to increased levels of SAH, a very potent inhibitor of several SAM‐dependent MTs. Other competitive SAM inhibitors (such as thiazolo‐pyrazole and pyrazolo‐pyrazole derivatives) have recently been developed that could be further optimised as new drugs useful for the treatment of Parkinson's disease, as adjuncts for levodopa (l‐DOPA)‐based therapies or the treatment of schizophrenia.^[109]^

COMT and other SAM‐dependent MTs have been shown to accept SAM analogues containing alternative *S*‐alkyl, ‐allyl, or ‐propargyl substituents, leading to unnatural alkylation reactions.^[79b]^ Czarnota *et al*., reported that sinefungin (adenosyl‐l‐ornithine), a fungal derived inhibitor of SAM‐dependent MTs, could be used as a transition state analogue of COMT when combined with a catechol.^[110]^ Furthermore, Herbert *et al*.^[111]^ used carboxy‐*S*‐adenosyl‐l‐methionine as an alternative cofactor to generate carboxymethylated and ‐ethylated products with interesting physicochemical properties and new biological activities and functions, due to the polar character of the carboxymethyl group.

The relaxed substrate and cofactor specificity of COMT was exploited by Struck *et al*.^[112]^ to label tyrosine residues with alkyl groups in peptides. They developed a strategy to selectively convert tyrosine‐containing peptides (e. g. linear synthetic peptides, human hormones, cyclic peptide antibiotics) to l‐DOPA by means of a fungal tyrosinase catalysed enzymatic hydroxylation followed by COMT‐mediated *O‐*alkylation. This methodology can also be used to label or capture proteins and peptides containing human l‐DOPA for biomedical diagnostic applications.

In addition to the appreciable substrate promiscuity, COMT has also potential as a biocatalyst in regiospecific alkylation reactions (Figure 8). In mammals, native COMTs have a clear *meta* selectivity, which is in accordance with positioning in the active site. Polar and ionisable substituents such as those found in catecholamines, COMT natural substrates, are more likely to orientate out of the active site and toward the solvent, resulting in *meta*‐methylation. In contrast, *para*‐methylation is more evident for substrates with neutral or more hydrophobic substituents that can be oriented toward the so‐called hydrophobic wall.^[113]^

Since both *para*‐ and *meta*‐methylated catechols are found in pharmaceuticals, ‐e. g. the anti‐inflammatory drug Piclamilast (*para*‐substituted) and Calebin A, a *meta*‐methylated drug used for the treatment of gastric cancers, COMT variants with improved regioselectivity were developed.^[114]^ The ability of these COMT variants to transfer alkyl groups regioselectively from ethyl, allyl, and benzyl SAM analogues to small molecules such as 3,4‐dihydroxybenzaldehyde, 4‐nitrocatechol, 3,4‐dihydroxyphenylacetic acid, 3,4‐dihydroxybenzoic acid or clinically important natural products like the immunosuppressive agents rapamycin has been exploited.^[115]^ The aforementioned enzymatic alkyl diversification offers an attractive route to generate new drug analogues.

**Figure 9:**
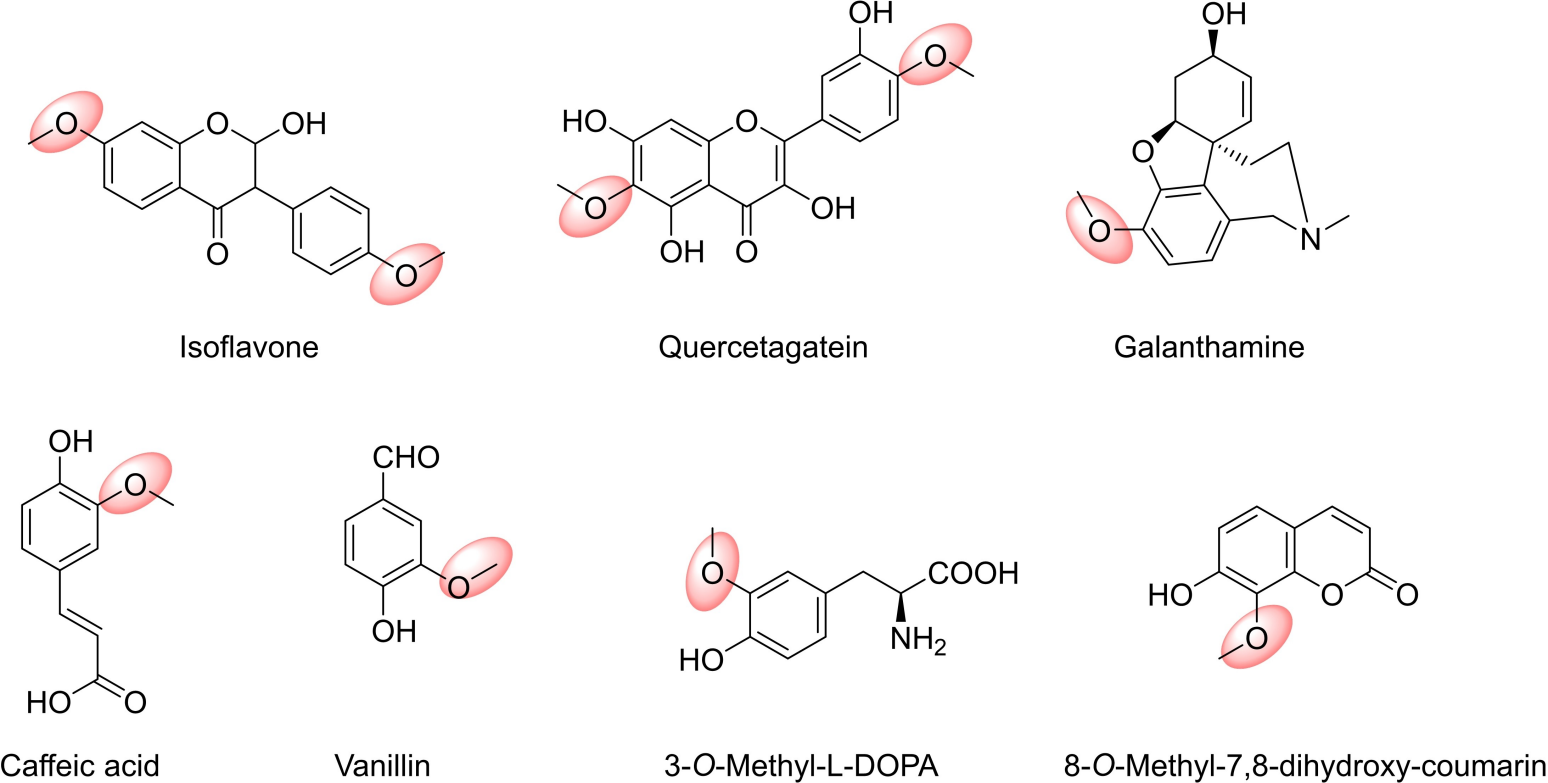
*O*‐Methyl transferases (OMTs) as catalysts are involved in the biosynthesis of aromatic compounds.

Improved *meta*‐selective COMT mutants have been used to increase vanillin production in yeast or *E. coli*. Vanillin (Figure 9), the world‘s most widely used food additive for flavouring and also an intermediate in the chemical and pharmaceutical industries for the production of herbicides, antifoaming agents and medicines, is naturally obtained from the tropical vanilla orchid (*Vanilla planifolia*). OMTs from plants are involved in the biosynthesis of aromatic compounds such as vanillin, but the inefficiencies derived from cultivation, in particular of *V. planifolia*, are manifested in the high price of natural vanilla extract.^[116]^ *De novo* production of vanillin in a heterologous host organism from glucose was first reported in both *S. cerevisiae* and *Schizosaccharomyces pombe*. The metabolic pathway designed had three heterologous enzymes: a dehydroshikimate dehydratase, an OMT, and a carboxylic acid reductase.^[117]^ A similar route was designed by Li and Frost^[118]^ in *E. coli*, which was recently optimised by Kunjapur *et al*.,^[119]^ implementing strategies to increase SAM availability, the limiting step of the biosynthetic route in *E. coli*.

Regioselectivity was also exploited to develop a real‐time assay of COMT activity based on the fluorescence of the 8‐*O‐*methyl derivative of 7,8‐dihydroxy‐coumarin. This methodology was successfully used to determine endogenous COMT activity in living cells and rat brain slices.^[56b]^

Plant OMTs are a particular group of enzymes, which show a great variety in their active sites, and accept as substrates a wide variety of alkaloids, flavonoids, lignols, phenylpropanoids, terpenoids and other diverse natural products. As a consequence, regioselectivity is also more varied in plants and depends on each particular substrate‐enzyme pair. There are however strict *meta*‐selective caffeoyl/5‐hydroxyferulolyl coenzyme A ester *O*‐MTs described in lignin biosynthesis,^[47b]^ as well as a *para*‐selective enzyme norbelladine 4′‐*O‐*methyltransferase (N4OMT) from *Narcissus sp. aff. pseudonarcissus* involved in the biosynthesis of galanthamine (GAL, Figure 9), that is used in the treatment of Alzheimer's.^[120]^

**Figure 10:**
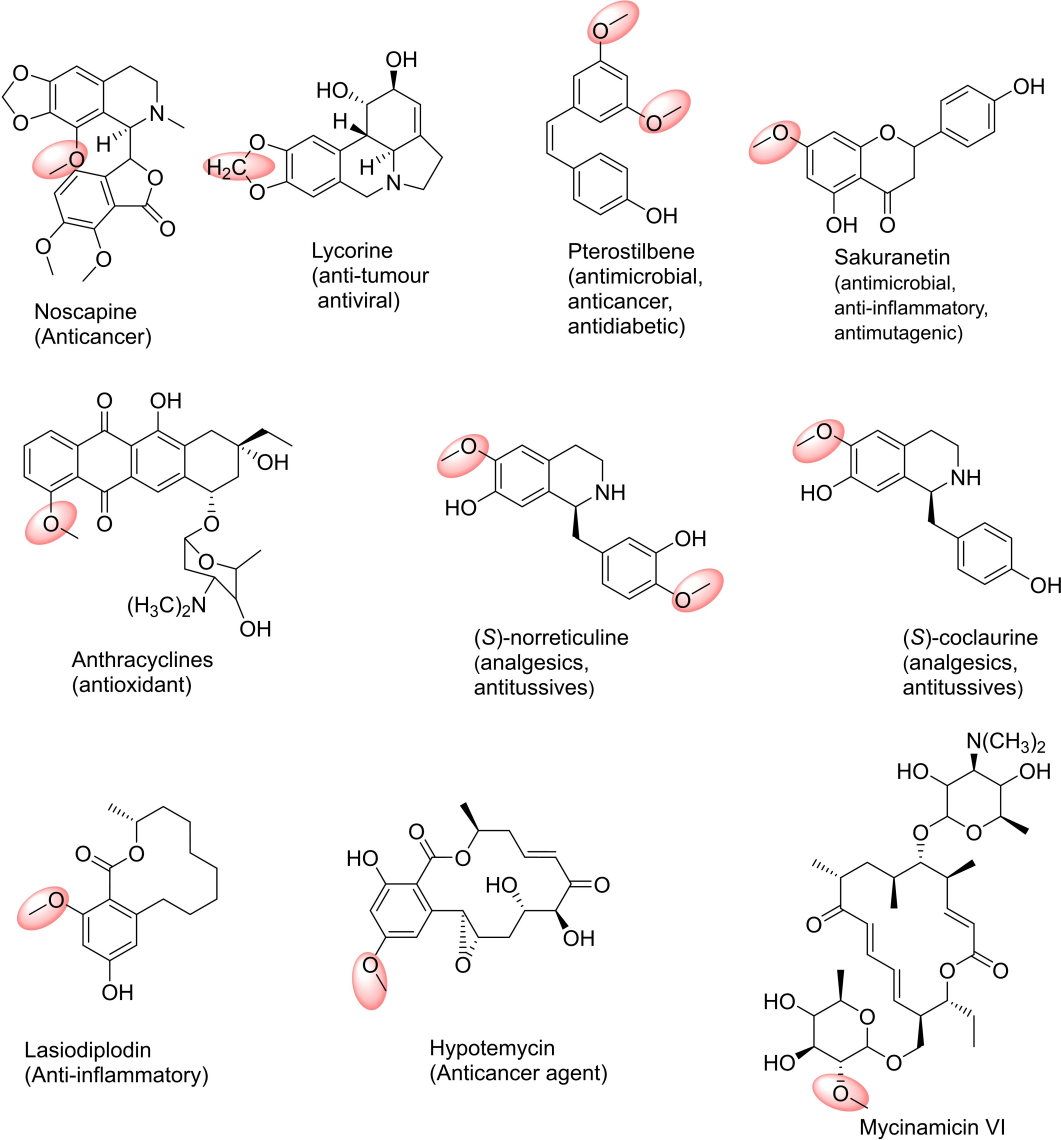
Examples for drug targets in the pharmaceutical industry: products of *O*‐methyl transferases (OMTs) catalysed reactions.

In addition to GAL, other *Amaryllidaceae* alkaloids, such as lycorine (Figure 10) and narciclasine, exhibit interesting anti‐tumour and antiviral activities. All of them are biosynthesised from the common intermediate 4′‐*O‐*methylnorbelladine that is formed by the *O‐*methylation of norbelladine catalysed by N4OMT. Recently, an OMT with high amino acid homology with NpN4OMT was isolated and characterised from *Lycoris aurea*. Unlike NpN4OMT, LaOMT1 showed *meta*‐*O*‐methylation preference towards 3,4‐dihydroxybenzaldehyde, caffeic acid and norbelladine.^[121]^ On the other hand, *Lycoris radiate* produces many types of Amaryllidaceae alkaloids such as GAL, lycorine, narciclasine, pseudolychorine, and hippacine, whose structures contain methoxy groups located at different positions. LrOMT was demonstrated to be a promiscuous OMT that can recognise aromatic compounds with at least two adjacent hydroxyl groups as substrates, and possess differential regioselectivity depending on the properties of the binding groups. Substrates with electron‐donating amine groups showed preference for *para*‐methylation, while *meta*‐methylation was observed on substrates with electron‐withdrawing groups like aldehyde or carboxyl.^[122]^

Recently, from structural studies of the *Papaver somniferum* OMT, PSMT1, which catalyses the 9‐*O‐*methylation of scoulerine, it was proposed that regioselectivity can be predicted from genomic resources.^[123]^ Methylation of scoulerine is the first step in noscapine biosynthesis in the latex of opium poppy (*P. somniferum*), an opiate that has no addictive properties. The recent *de novo* production of noscapine and a number of halogenated derivatives in *S. cerevisiae* opens the way towards the development of noscapine and its analogues as potential drug leads.^[124]^

Unlike plants, OMTs from microorganism sources have been less studied despite their ability to methylate a wide variety of natural plant products. COMT‐like enzymes with varied regioselectivity are also found in bacteria, although *para*‐methylation is generally favoured, especially with natural substrates. As an example, MxSafC, from the saframycin biosynthetic pathway in *Myxococcus xanthus*, is strictly *para* selective for DOPA, its natural substrate, while for 3,4‐dihydroxybenzoic acid it showed a strong *meta* preference.^[124]^

Different *Streptomyces* OMTs were found to be useful in the methylation of natural products such as flavonoids, chalcones, anthraquinones, anthracyclines and sterols, providing compounds with interesting biological properties. In particular, SpOMT from *S. peucetius* showed the possibility of *O‐*methylating various flavonoids and anthraquinones, among them 7‐hydroxy‐8‐methoxy flavone, which has demonstrated long‐term cytoprotective and antioxidant effects compared to 7,8‐dihydroxyflavone.^[125]^ SaOMT‐2 from *S. avermitilis* MA‐4680 showed high selectivity in the methylation of the 7‐hydroxy position of isoflavones, flavones and flavanones and ScOMT1 from *S. coelicolor* A3 (2) showed preference for the methylation of *ortho*‐dihydroxyflavones.^[126]^

Sakuranetin (Figure 10), the main flavonoid phytoalexin in rice, is also highly valuable to the nutraceutical and pharmaceutical markets since in addition to its antimicrobial activity, it showed anti‐inflammatory, antimutagenic, anti‐*Helicobacter pylori*, antileishmanial, and antitrypanosomal properties. Wang *et al*.^[127]^ designed two strains of *E. coli* working in tandem to reconstitute the sakuranetin biosynthesis pathway, overall containing seven heterologous enzymes. In the upstream strain, *p*‐coumaric acid was obtained from glucose, which was then used as a substrate for the downstream strain to produce naringenin. The last step in this co‐culture biosynthetic system was the *O‐*methylation of naringenin by naringenin 7‐OMT from *Oryza sativa* to afford the sakuranetin product with high efficiency.

Kallscheuer *et al*.^[128]^ demonstrated that *Corynebacterium glutamicum* can be an alternative host to *E. coli* and *S. cerevisiae* to synthesise several different polyphenols such as stilbenoids and flavonoids. The introduction of a heterologous resveratrol‐di‐*O‐*MT from *Vitis vinifera* (common grape vine) into a resveratrol‐producing *C. glutamicum* strain allowed the synthesis of mono‐*O‐*methylated pinostilbene and di‐*O‐*methylated pterostilbene from *p*‐coumaric acid. Methylation increased the antimicrobial, anticancer, or antidiabetic activities of such stilbene compounds by increasing solubility, stability, or absorption in human cells.

Unlike bacterial OMTs, little has been explored regarding the substrate promiscuity and regioselectivity of secondary metabolite fungal OMTs. Benzenediol lactones (BDL) are an interesting group of fungal polyketides natural products having a variety of biological activities; their methylation, carried out by fungal OMTs, is crucial in modulating these activities. Using LtOMT of *Lasiodiplodia theobromae*, lasiodiplodin, an inhibitor of prostaglandin and ATP synthesis, can be obtained by methylation of the phenolic hydroxyl C‐3 of the resorcyl acid lactone desmetillasiodiplodin.^[129]^ HsOMT (Hpm5) from *Hypomyces subiculosus* methylates the phenolic hydroxyl C‐5 of the resorcyl acid lactone trans‐14,15‐dehydrozearalenol, to provide a methylated intermediate of hypotemycin, a selective inhibitor of mitogen‐associated protein kinases.^[130]^ Studies carried out by Wang *et al*.^[131]^ showed that wild‐type LtOMT and HsOMT are regiospecific enzymes; LtOMT affords *ortho*‐methylation and HsOMT *para*‐methylation with respect to the aromatic carbon carrying the carbonyl group. In addition, both enzymes showed broad substrate flexibility, methylating a wide collection of natural and unnatural BDLs, such as resorcyl and dihydroxyphenylacetic acid lactones, with variations in size or functionalisation, and some non‐macrocyclic BDL congeners such as isocoumarins. In the biosynthesis of the macrolide antibiotic mycinamicin two methylations at two positions on the 6‐deoxyallose substituent occur. The methylations are catalysed by MycE, 2′‐OMT and then MycF, 3′‐OMT to produce the mycinose moiety of the final product.[^106b^, ^132^] In other work, catechol *O*‐MTs from *Rattus norvegicus* (*Rn*COMT), *Coptis japonica* (*Cj*‐6‐OMT) and *Mx*SafC and coclaurine *N‐*MT (CNMT) were used in successful methylations for the structural diversification of 12 tetrahydrosoquinolines (THIQs). The SAM supply and SAH degradation three‐enzyme cascade (*Ec*MAT and *Ec*MTAN) were used with the MTs for scaling the reaction up to determine product regioselectivities. Interestingly, the regioselectivities of the MTs were dependent on the C‐1 group and presence of fluorine in the THIQ. Dual activity was also noted when using the substrate norlaudanosoline where *Rn*COMT and *Mx*SafC could regioselectively methylate two different catechol rings giving products such as (*S*)‐norreticuline (Figure 10). A seven‐enzyme cascade was also constructed with a tyrosinase, decarboxylase, transaminase and norcoclaurine synthase to construct the THIQ, with subsequent addition of the MTs, highlighting their applications in *in vitro* pathways to new THIQs. In related work *Rn*COMT and *Mx*SafC were used in the regioselective methylation of THIQs with phenyl and cyclohexyl groups at C‐1. Here the same regioselectivity was observed with both MTs, with C‐6‐OMe products formed preferentially. Enantioselectivity for *Mx*SafC towards the (1*S*)‐THIQs was also noted.^[133]^ Other applications of *Rn*COMT and *Mx*SafC include their use in routes towards tetrahydroberberine and protoberberine alkaloids, for regioselective C‐6‐OH methylations of THIQ scaffolds.^[134]^

#### 5.1.1 *N*‐Methyltransferases (NMTs)

The second class of NPMTs is natural product *N*‐MTs (NMTs) which are widely represented across all domains of life. Natural product NMTs are not as numerous as OMTs; nevertheless, they are widespread in living organisms, being responsible for the methylation of a wide variety of compounds such as peptides, glycosamines, linear and heterocyclic amines (e. g. indoles, imidazoles, and alkaloids).^[43]^

Phenylethanolamine *N‐*MT (PNMT) naturally catalyses the methylation of norepinephrine to adrenaline, using SAM as a methyl donor. This reaction represents the terminal step in catecholamine biosynthesis.^[135]^ Human PNMT and its product, adrenaline, are involved in various processes of the central nervous system: cardiovascular homeostasis, activation of the adrenergic receptor, learning and memory, and regulation of the circadian cycle. Different studies about the human PNMT demonstrate that this enzyme is important in various human diseases such as hypertension, Alzheimer's and Parkinson's.^[136]^ Grunawald *et al*.^[137]^ showed the influence of substrate structure and stereochemistry on enzyme activity. Only substrates such as phenylethanolamine and *cis*‐2‐amino‐tetralol could be methylated since the simultaneous binding of the amino and hydroxyl groups are required for the activity of the PNMT. Following this, methylation of octopamine, a phenyl ethanolamine analogue having an additional hydroxyl group, was recently reported. The directed evolution of PNMT carried out by Luo *et al*.^[138]^ produced a PNMT variant that can methylate octopamine to produce synephrine with 2‐fold activity improvement.

**Figure 11:**
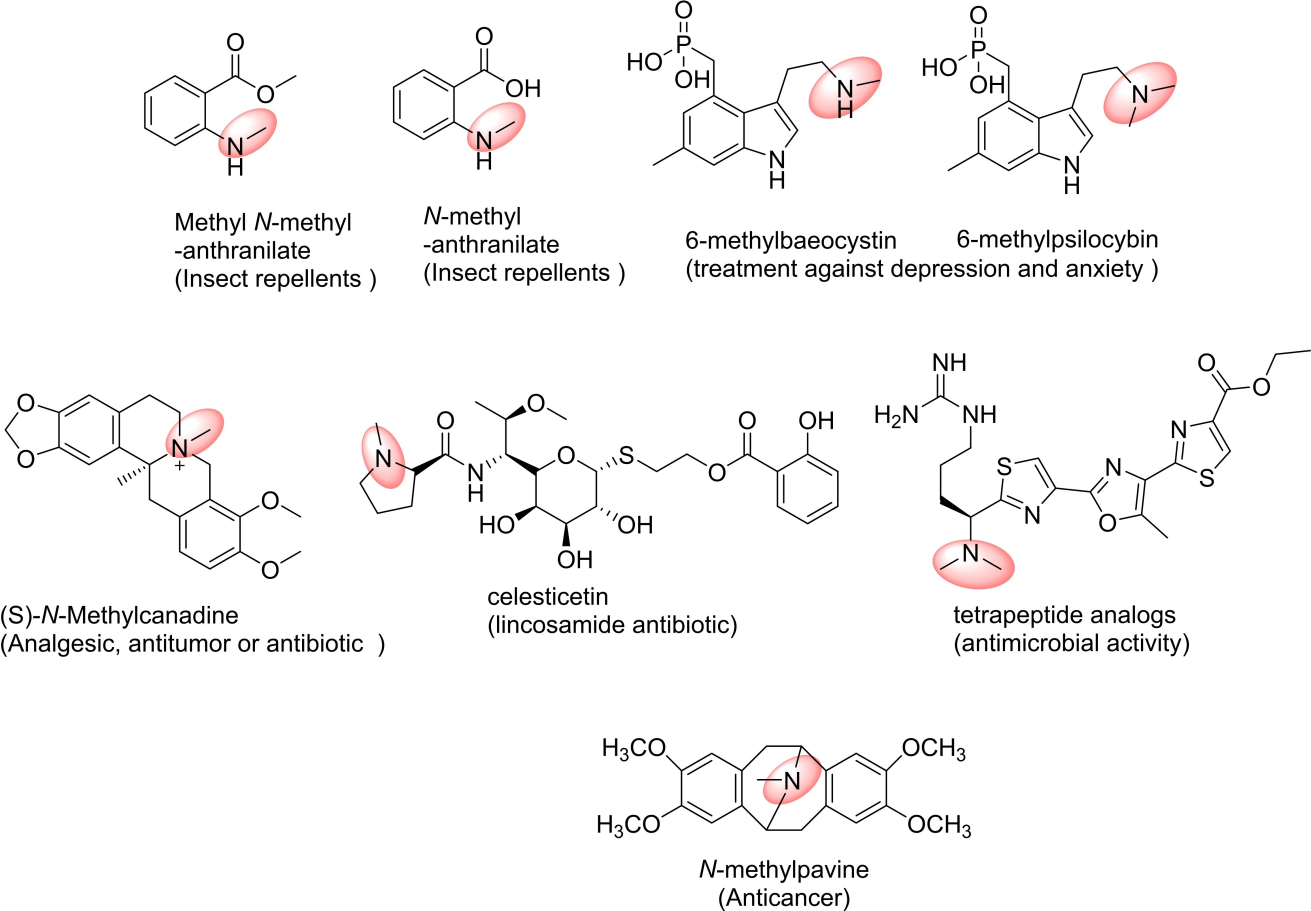
Examples for pharmaceuticals compounds: products of *N*‐methyltransferases (NMTs) catalysed reactions.

Anthranilic acid (2‐aminobenzoic acid) is an intermediate for tryptophan and alkaloid biosynthesis and a precursor of a wide range of compounds, including flavours and fragrances, pharmaceuticals, and insect repellents (Figure 11).^[139]^ Lee *et al*. synthesised three anthranilate derivatives: *N‐*methylanthranilate, ethyl *N‐*methylanthranilate and methyl *N‐*methyl‐anthranilate from glucose, using engineered *E. coli* cells containing *N‐*MT from *Ruta graveolens*, anthraniloyl‐coenzyme A (CoA) : methanol acyltransferase from *Vitis labrusca*, and anthranilate coenzyme A ligase from *Pseudomonas aeruginosa*.^[140]^

Pseudoephedrine and ephedrine have been synthesised employing two *E. coli* strains with expression vectors containing genes encoding a ω‐transaminase from *Pseudomonas putida* and a phenylalkylamine NMT (PaNMT) from *Ephedra sinica*.^[141]^ A co‐culture of these strains produced pseudoephedrine or ephedrine from (*R*) or (*S*)‐phenylacetylcarbinol, respectively, through the *N‐*methylation of the corresponding intermediates norpseudoephedrine and norephedrine. Other naturally occurring phenylalkylamines, including cathinone and pseudoephedrine along with several tetrahydroisoquinolines and benzylisoquinolines were also accepted as PaNMT substrates.

Benzylisoquinoline alkaloids have a range of pharmacological properties, which can be modulated by methylation using CNMT.^[142]^ Recently, the use of recombinant CNMT from *Coptis japonica* was demonstrated on twenty substrates, including coclaurine, heliamine, norcoclaurine, and many C‐1 substituted THIQs affording *N‐*methylated products in high yield. In addition, substituted SAM analogues were also accepted by CNMT, leading to different *N‐*alkylated products.^[7a]^

The indole alkaloids psilocibin and baeocystin are used as treatments for depression and anxiety. Their methylated derivatives, 6‐methylpsilocibin and 6‐methylbaeocystin have been synthesised from 4‐hydroxy‐6‐methylindole through the biosynthetic route proposed by Fricke *et al*.^[143]^ In the first step, 4‐hydroxy‐6‐methylindole was converted into 4‐hydroxy‐6‐methyl‐l‐tryptophan by tryptophan synthetase from *Salmonella enterica*. Then, two enzymes from *Psilocybe cubensis* – decarboxylase and kinase – produced 6‐methylbaeocystin. Finally, a SAM dependent NMT from *P. cubensis* was engineered to produce 6‐methylbaeocystin and 6‐methylpsilocybin.

#### 5.1.2 *C*‐ and *S*‐methyltransferases (CMTs and SMTs)

*C*‐directed MTs and *S*‐directed MTs are less common. The known CMTs participate in specialised metabolic reactions and methylate reactive aromatic compounds such as phenols and tetrapyrroles, and also activated aliphatic compounds. TcaB9 for example from *Micromonospora chalcea* functions as a C‐3′‐MT involved in the production of d‐tetronitrose.^[144]^

Small molecule *C‐*MTs (CMTs) are found more frequently in bacterial and plant systems, but have not yet been detected in humans and are rare in other eukaryotes.^[38b]^ *C–*Methylation is a frequently‐used strategy in the pharmaceutical industry for optimising drug leads.^[145]^ There are many described CMTs from *Streptomyces* that could be applied in organic synthesis.^[146]^ For example, CMT from *Streptomyces rishiriensis* CouO is implicated in the biosynthesis of the antibiotic coumermycin A1. Moreover, this enzyme can catalyse the methylation of *N‐*(4,7‐dihydroxy‐2‐oxo‐2*H*‐chromen‐3‐yl)benzamide, a curcumin compound.^[147]^ SgvM CMT from *Streptomyces griseoviridis*, which is part of the octadepsipeptide antibiotic viridogrisein biosynthesis, can also methylate or dimethylate 2‐oxovalerate, pyruvate, 2‐oxobutyrate and phenylpyruvate.[^148^, ^149^] On the other hand, CMT NovO from *Streptomyces spheroids*, involved in the biosynthesis of the antibiotic novobiocin catalyses the methylation of aminocoumarins and hydroxy‐coumarins such as 4,5,7‐trihydroxy‐3‐phenyl‐coumarin in the C‐8 position and also naphthalenediols.^[150]^

**Figure 12:**
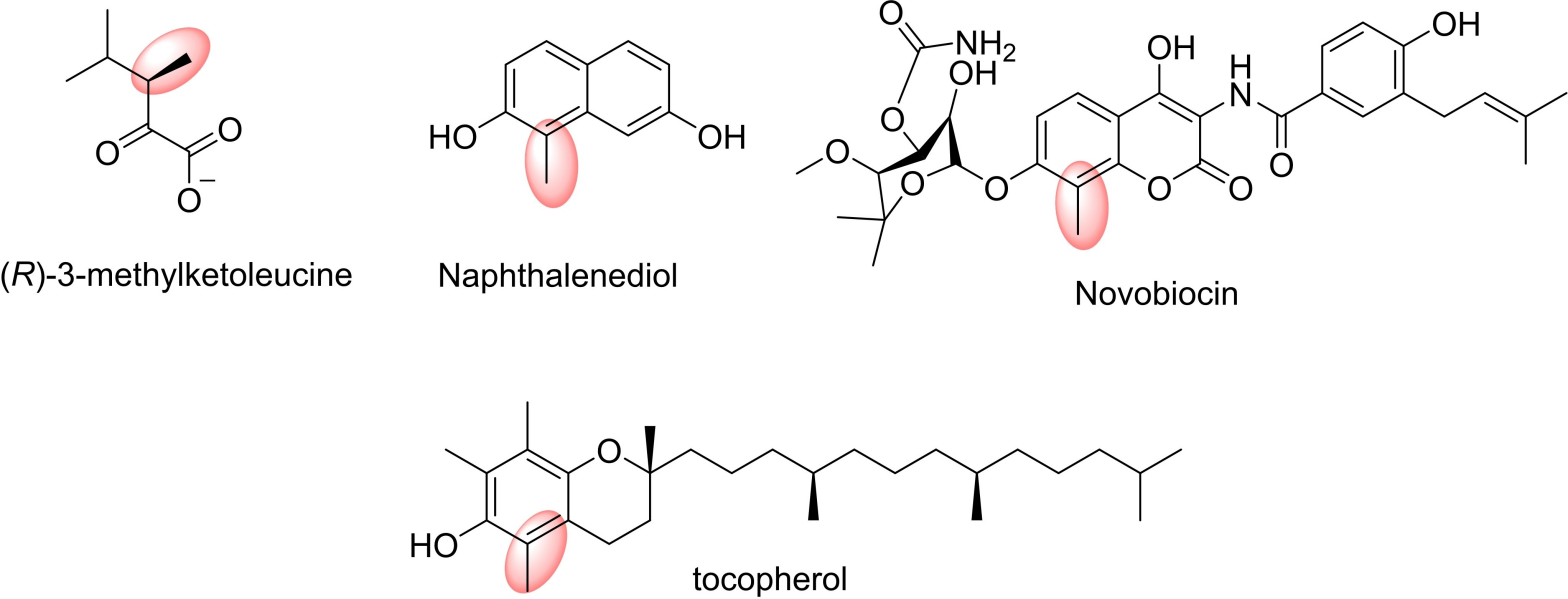
Examples for pharmaceuticals compounds: products of *C*‐methyltransferases (CMTs) catalysed reactions.

Thiopurine *S*‐MT (TPMT), an SMT in thiopurine prodrugs synthesis catalyses the methylation of 6‐mercaptopurine.^[151]^ Also, *C. roseus S*‐MT 1 (CrSMT1) has been used to methylate a wide range of aliphatic and aromatic sulfhydryl substrates (Figure 12).^[152]^

## 6 Conclusion and Outlook

SAM‐dependent MTs are versatile enzymes that display excellent chemoselectivities allowing methyl groups to be added selectively to S, N, O, or C atoms. They equally show high regioselectivity in molecules with multiple functional groups, for example, distinguishing between different hydroxyl groups in polyphenols and sugars. To date the range of enzymes is however still limited, in particular for aliphatic substrates. At the same time these are the substrates that require all types of selectivity.

Nevertheless, there are wide potential applications of SAM and in recent years significant steps towards developing SAM to introduce other alkyl groups have been made. For this, MTs have been turned into alkyltransferases and in the future much more can be expected here. A drawback at this time remains: a reliable regeneration system is required that does not use toxic reagents to introduce the methyl/alkyl groups. While MTs introduce the desired selectivity, greatly reducing waste in the synthesis of products, toxic methyl iodide should ideally be replaced by a sustainable and benign reagent to recycle SAM. Given the major steps made in recent years, it can however be expected that such challenges will also be embarked upon in the future. Methionine available from fermentations can also be utilised for SAM production as detailed above. With such developments, the potential of MTs in biology will be transferred to the laboratory and the clinic, through the production of APIs in a selective and sustainable manner.

## Conflict of interest

The authors declare no conflict of interest.

## Biographical Information

*Elizabeth Lewkowicz studied Chemistry at Universidad de Buenos Aires, and obtained her PhD degree in Organic Chemistry in 1990. She acquired training in Biocatalysis during her postdoctoral stay at the Department of Organic and Pharmaceutical Chemistry of the Faculty of Pharmacy at Universidad Complutense de Madrid, Spain. Since 1992 she has been working in the Laboratory of Biotransformations and Nucleic Acid Chemistry at the Universidad Nacional de Quilmes, Argentina as full Professor. In 2004 she became a member of the National Research Council of Argentina (CONICET). Her research interests focus on the development of biocatalytic and organocatalytic strategies for the preparation of nucleoside derivatives*.

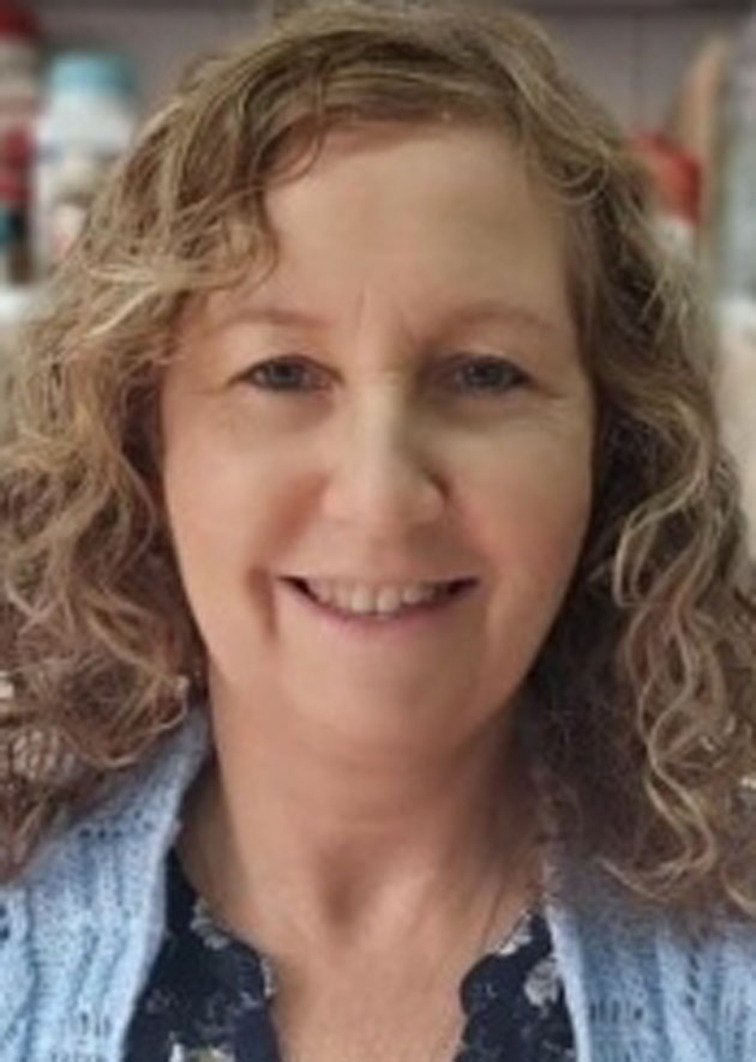

## Biographical Information

*Romina Fernandez Varela received her degree in Biotechnology in 2016 at the Universidad Nacional de Quilmes, Argentina. She is now pursuing her PhD under the supervision of Prof. Elizabeth Lewkowicz in the Laboratory of Biotransformations and Nucleic Acid Chemistry. Her current research interests include the application of enzymes in the synthesis of chiral compounds of chemical and pharmacological importance*.

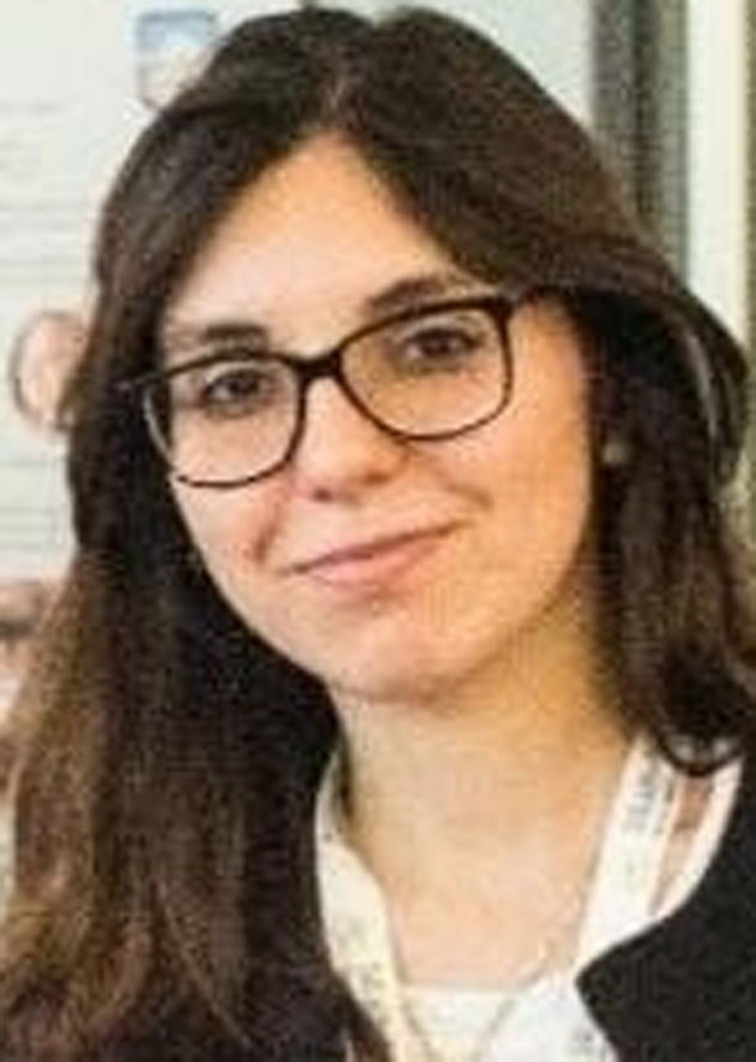

## Biographical Information

*Adolfo Iribarren received his PhD in Chemistry from the University of Buenos Aires, Argentina. He conducted his postdoctoral studies at the European Molecular Biology Laboratory (EMBL, Heidelberg, Germany) as an Alexander von Humboldt Fellow and later as a staff member. He then moved to the Molecular Biology Research Institute (IRBM‐Rome, Italy), as group leader of the Nucleic Acid Chemistry Laboratory. Upon his return to Argentina he founded the Laboratory of Biotransformations and Nucleic Acid Chemistry at the University of Quilmes, where he is currently a Professor and Researcher of CONICET (National Research Council of Argentina)*.

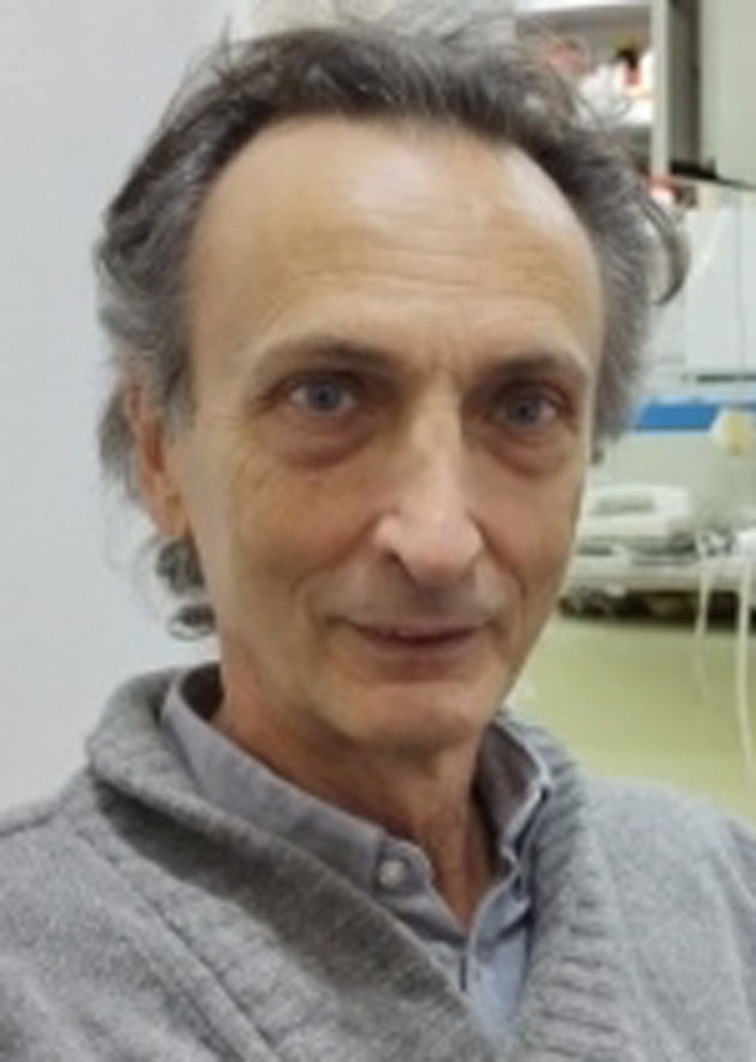

## Biographical Information

*Ben Thair completed his undergraduate degree in Molecular Genetics at King's College London, with an extra mural year in the laboratory of Professor Julie Ahringer at The Gurdon Institute, Cambridge. He is currently in the Wellcome Trust Structural, Computational and Chemical Biology programme at the Institute of Structural and Molecular Biology, under the supervision of Professors Helen Hailes and John Ward. His work focusses on developing enzyme cascades for the functionalisation of pharmacologically relevant scaffolds*.

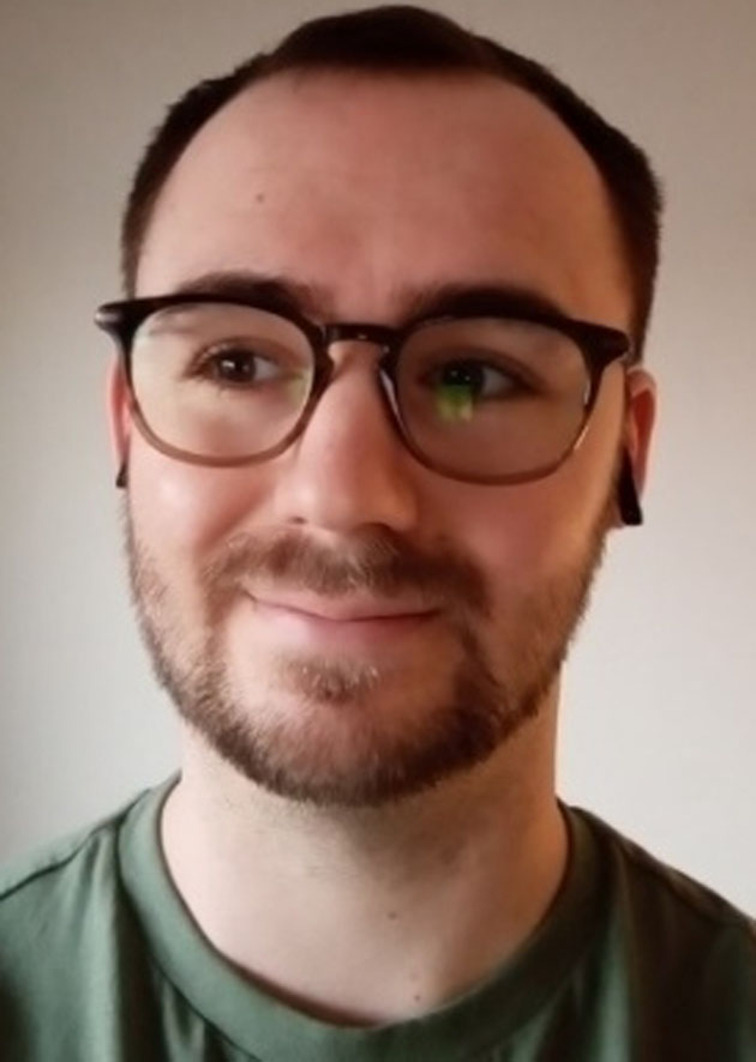

## Biographical Information

*Helen Hailes is Professor of Chemical Biology in the Department of Chemistry at University College London. She is interested in developing sustainable synthetic methods using biocatalysts in single‐step reactions, multistep enzymatic or chemoenzymatic cascades. A recent focus has also been the use of biomass waste, as a sustainable feedstock to produce higher value compounds. As well as the use of enzymes for synthetic applications, investigations also involve the discovery and use of enzymes for the degradation of plastics and other waste materials for molecular recycling*.

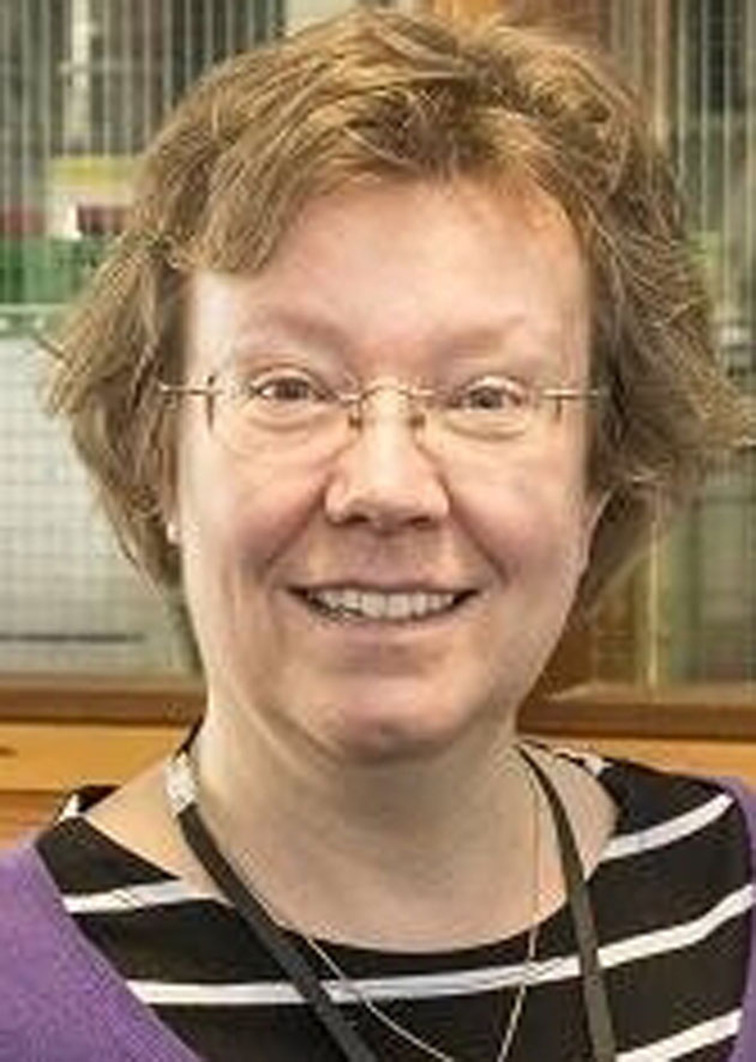

## Biographical Information

*John Ward has a BSc in Biochemistry from the University of Bristol and he received his PhD from the Microbiology Department in Bristol under Prof John Grinsted. He carried out postdoctoral research on antibiotic resistance plasmids and transposons and then moved to UMIST in Manchester to work on the TOL plasmid meta‐cleavage pathway. He moved to UCL, London, as a lecturer in the Biochemistry Department and in 2012 moved to the Biochemical Engineering Department at UCL where he works on biocatalysis, metagenomics and biodegradation of plastics*.

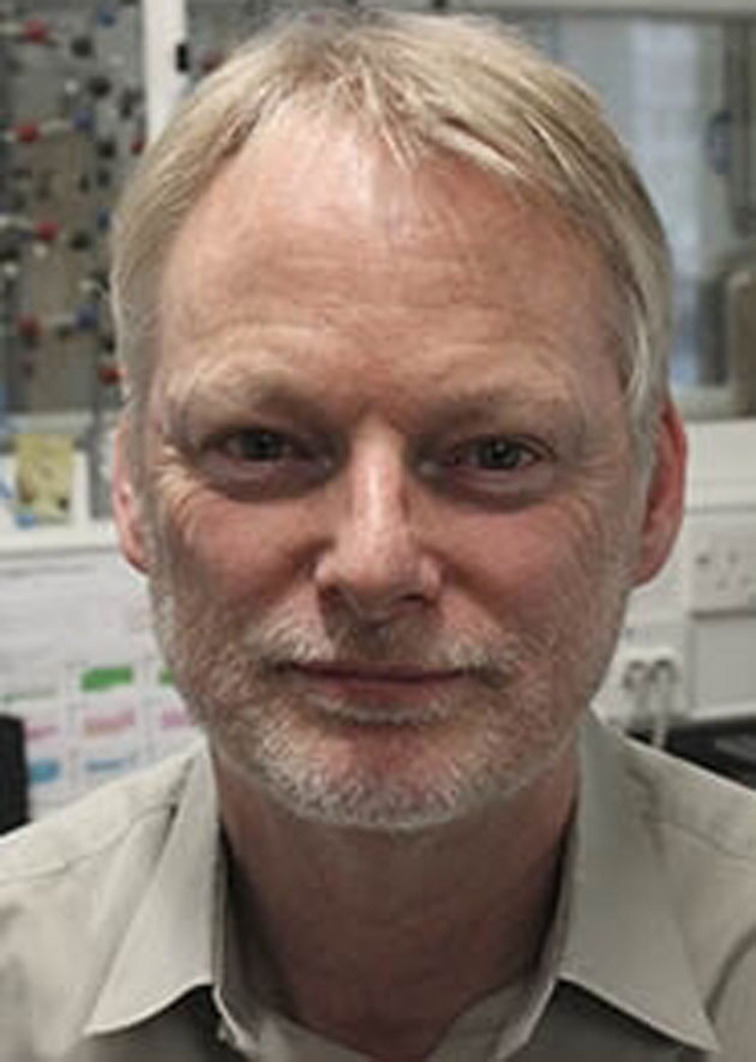

## Biographical Information

*Eman Abdelraheem received her Master's degree in chemistry from Sohag University, Egypt. After working as an assistant lecturer at the same university until 2013, she started her Ph.D. studies with Prof. Alexander Dömling in the Institute of Pharmacy at the University of Groningen, the Netherlands. After obtaining her doctoral degree in 2018, she joined the Biocatalysis group at the Delft University of Technology working on multi‐enzyme cascade reactions under the guidance of Prof. Ulf Hanefeld*.

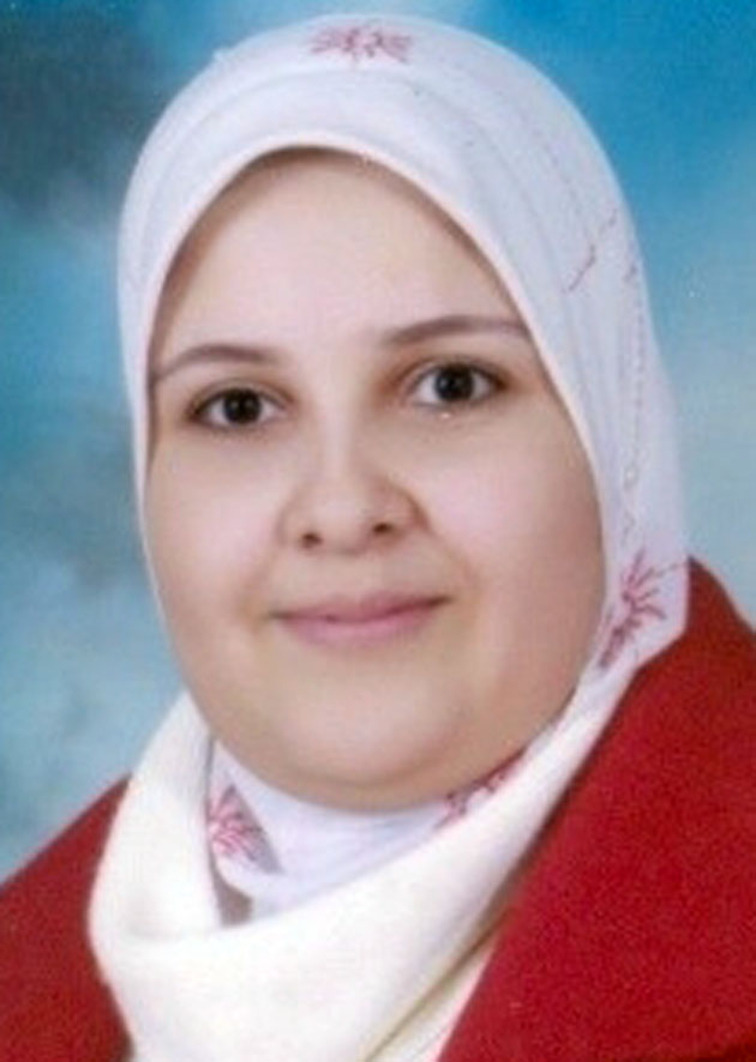

## Biographical Information

*Peter‐Leon Hagedoorn obtained his MSc degree Molecular Sciences in 1998 (Wageningen University, The Netherlands) after a short stay (1997–1998) in the laboratory of Prof. Michael Johnson (University of Georgia, Athens, USA). In 2002 he completed his PhD thesis on with Prof. Fred Hagen (Delft University of Technology in the Netherlands). He worked as a postdoctoral fellow (2002–2003) at Leiden University (The Netherlands) and subsequently (2003–2007) at Delft University of Technology. In 2007 he became an assistant professor in biocatalysis at the Delft University of Technology. His research is focussed on the elucidation of reaction mechanisms of (metallo‐)enzymes and metalloproteomics*.

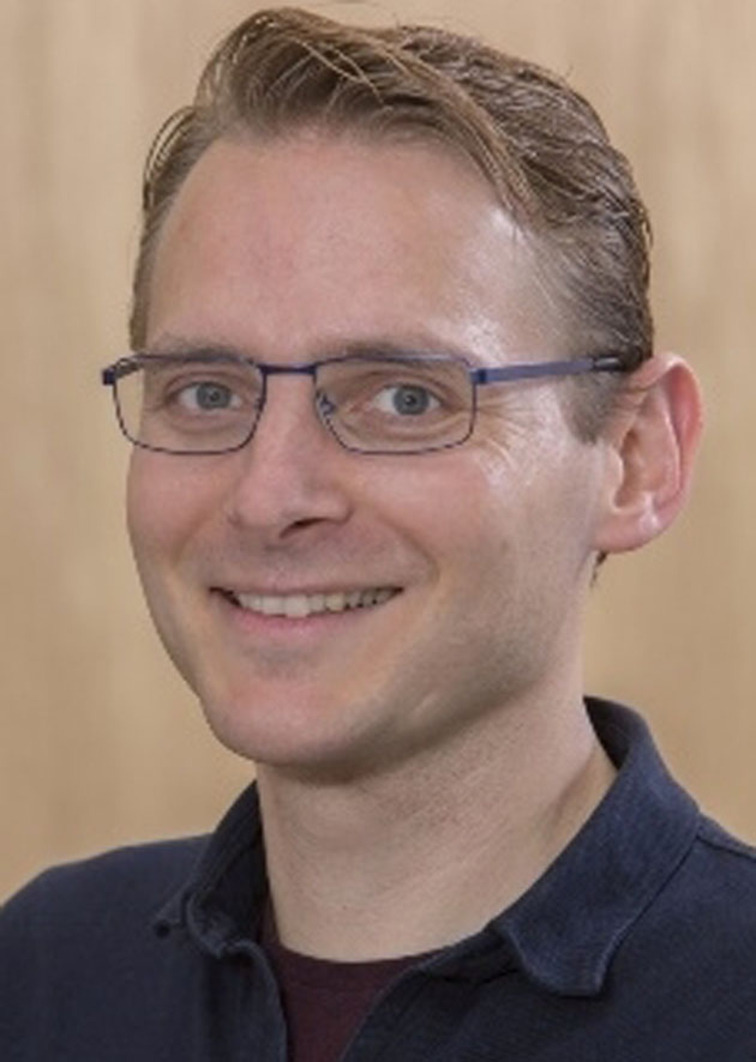

## Biographical Information

*Ulf Hanefeld received his PhD from the Georg‐August‐Universität zu Göttingen, having performed research both with Prof. H. Laatsch (Göttingen) and Prof. H. G. Floss (Seattle). After postdoctoral years with Prof. C. W. Rees (Imperial College London), Prof. J. Staunton (Cambridge) and Prof. J. J. Heijnen and Dr. A. J. J. Straathof (TU Delft), he received a fellowship from the Royal Netherlands Academy of Arts and Sciences (KNAW). His research in Delft focuses on enzymes, their immobilisation and application in organic synthesis*.

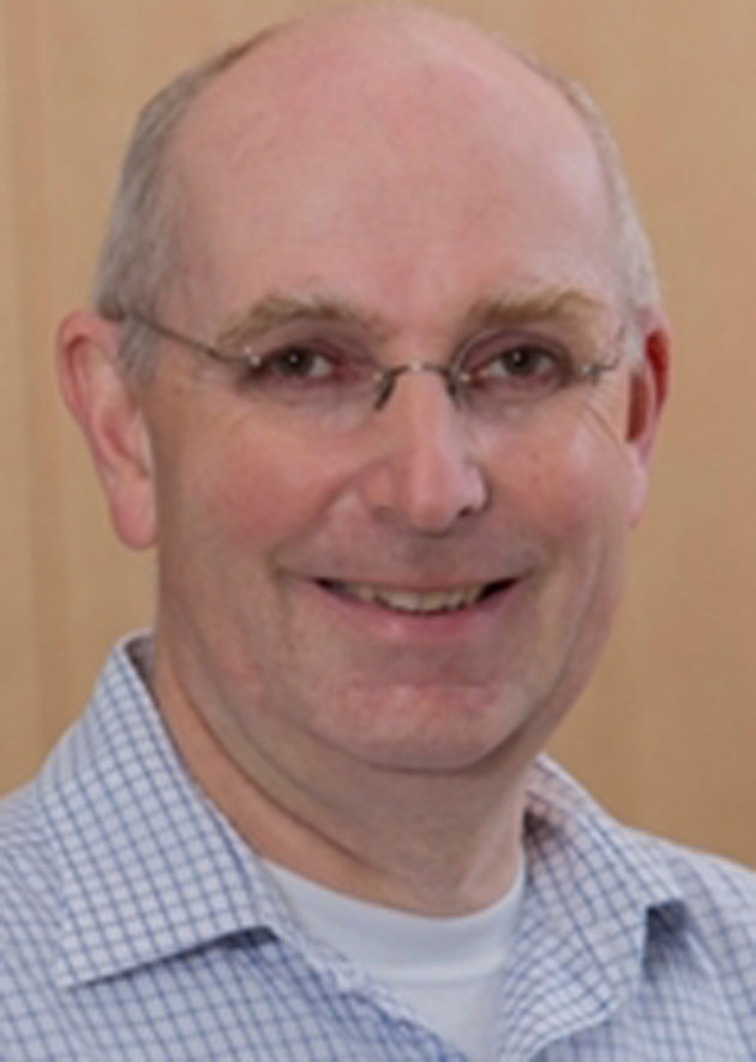

## Biographical Information

*Désirée Popadić studied Pharmaceutical Sciences at the University of Freiburg, Germany. During her Master's degree she spent a few months in the Truman group at the John Innes Centre in Norwich, UK, working on biosynthetic pathways in Streptomyces. Following a Master's thesis in Pharmaceutical Biology, she carried out her PhD in the Andexer group working on SAM‐dependent enzymes and SAM regeneration*.

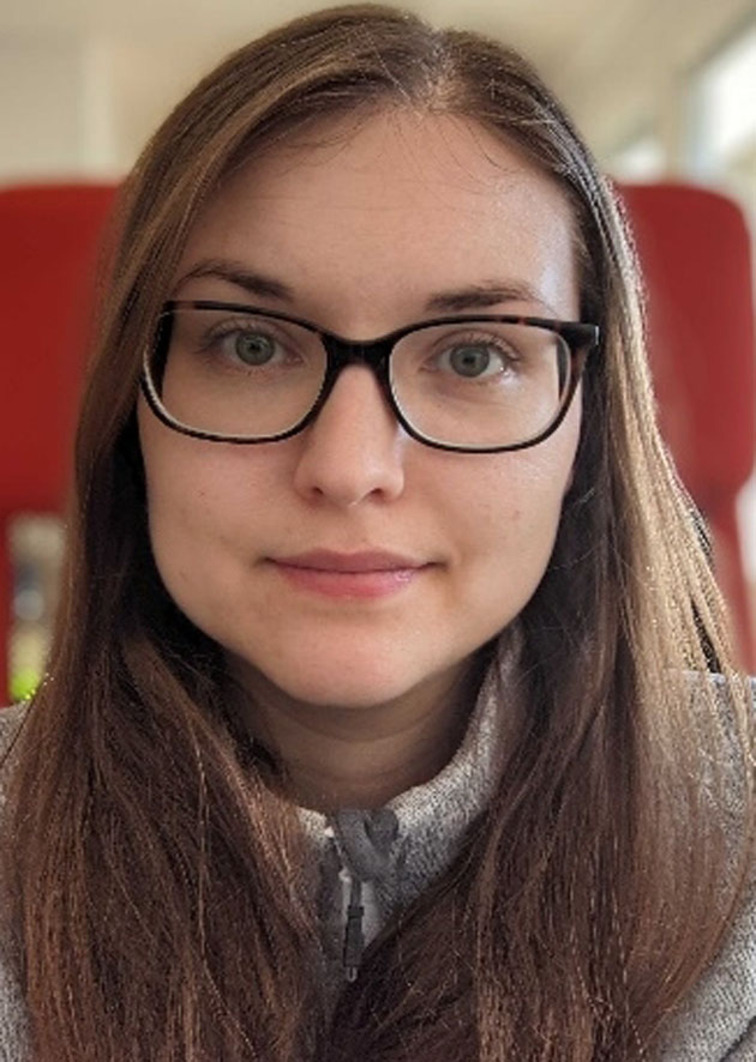

## Biographical Information

*Emely Jockmann is a PhD student in the department of Pharmaceutical Chemistry at the University of Freiburg, Germany. She graduated as a diploma pharmacist at the University of Freiburg. Her research interests focus on stereo‐ and chemoselective methylation reactions*.

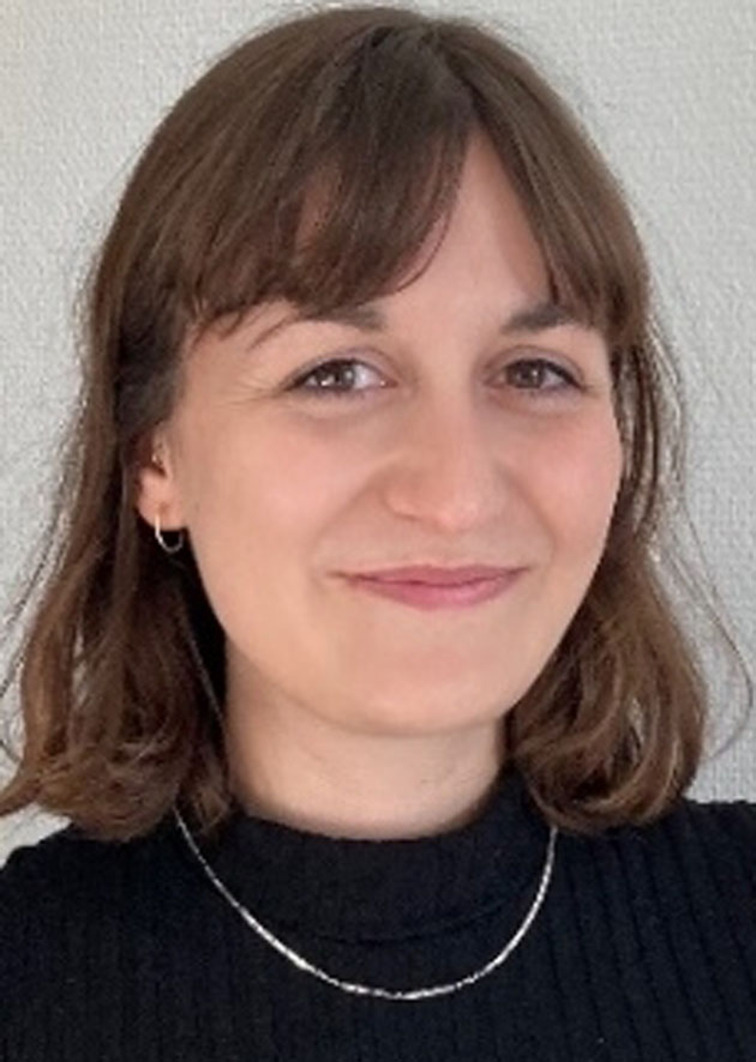

## Biographical Information

*Jennifer Andexer carried out her doctoral research working on novel hydroxynitrile lyases at the Institute of Molecular Enzyme Technology (University of Duesseldorf/Research Centre Juelich). In 2008, she moved to the Leadlay group at the University of Cambridge, and worked on the biosynthetic pathways of different natural products. She has been head of the Chemical Biology group at the Institute of Pharmaceutical Sciences (University of Freiburg) since 2011, and appointed Heisenberg professor for Pharmaceutical and Medicinal Chemistry in 2020. She works on the characterisation of cofactor‐dependent enzymes, cofactor regeneration systems and cofactor analogues*.